# Relative contribution of biomedical, demographic, and socioeconomic factors to COVID-19 vaccine receipt in rural India

**DOI:** 10.1371/journal.pone.0305819

**Published:** 2024-06-24

**Authors:** Bethany F. Ferris, Suganthi Balasubramanian, Anuradha Rajamanickam, Saravanan Munisankar, Bindu Dasan, Pradeep A. Menon, P'ng Loke, Subash Babu, Goylette F. Chami

**Affiliations:** 1 Nuffield Department of Population Health, Big Data Institute, University of Oxford, Oxford, Oxfordshire, United Kingdom; 2 National Institutes of Health – National Institute for Research in Tuberculosis -International Center for Excellence in Research (NIH-ICER), Chennai, Tamil Nadu, India; 3 Indian Council of Medical Research – National Institute for Research in Tuberculosis, Chennai, Tamil Nadu, India; 4 Laboratory of Parasitic Diseases, National Institutes of Health National Institutes of Allergy and Infectious Diseases (NIAID), Bethesda, Maryland, United States of America; University of Hyderabad, INDIA

## Abstract

**Background:**

In the first year of roll-out, vaccination for severe acute respiratory syndrome coronavirus 2 (SARS-CoV-2) prevented almost 20 million deaths from coronavirus disease 2019 (COVID-19). Yet, little is known about the factors influencing access to vaccination at the individual level within rural poor settings of low-income countries. The aim of this study was to examine determinants of vaccine receipt in rural India.

**Methods:**

A census of a rural village in Tamil Nadu was undertaken from June 2021 to September 2022. We surveyed 775 participants from 262 households. Household-level data on socioeconomic status (SES), water, sanitation, and hygiene practices, and individual-level demographic information, travel history, and biomedical data, including anthropometry, vital signs, and comorbidities, were collected. Logistic regression models with 5-fold cross-validation were used to identify the biomedical, demographic, and socioeconomic determinants of vaccine receipt and the timing of receipt within the first 30 days of eligibility. Vaccine ineligible participants were excluded leaving 659 eligible participants. There were 650 eligible participants with complete biomedical, demographic, and socioeconomic data.

**Results:**

There were 68.0% and 34.0% of individuals (N = 650) who had received one and two vaccine doses, respectively. Participants with household ownership of a permanent account number (PAN) or ration card were 2.15 (95% CI:1.32–3.52) or 3.02 (95% CI:1.72–5.29) times more likely to receive at least one vaccine dose compared to households with no ownership of such cards. Participants employed as housewives or self-employed non-agricultural workers were 65% (95% CI:0.19–0.67) or 59% (95% CI:0.22–0.76) less likely to receive at least one vaccine dose compared to salaried workers. Household PAN card ownership, occupation and age were linked to the timing of vaccine receipt. Participants aged ≤18 and 45–60 years were 17.74 (95% CI:5.07–62.03) and 5.51 (95% CI:2.74–11.10) times more likely to receive a vaccine within 30 days of eligibility compared to 19-44-year-olds. Biomedical factors including BMI, vital signs, comorbidities, and COVID-19 specific symptoms were not consistently associated with vaccine receipt or timing of receipt. No support was found that travel history, contact with COVID-19 cases, and hospital admissions influenced vaccine receipt or timing of receipt.

**Conclusion:**

Factors linked to SES were linked to vaccine receipt, more so than biomedical factors which were targeted by vaccine policies. Future research should explore if government interventions including vaccine mandates, barriers to vaccine access, or peer influence linked to workplace or targeted vaccine promotion campaigns underpin these findings.

## Introduction

Vaccination for severe acute respiratory syndrome coronavirus 2 (SARS-CoV-2) is estimated to have prevented almost 20 million deaths from coronavirus disease 2019 (COVID-19) in the first year of vaccine roll-out [1]. In low-income countries, 45% of deaths could have been prevented if the 20% vaccination coverage target set by the COVID-19 Vaccines Global Access (COVAX) had been met [1]. This figure rises to nearly all deaths if the 40% vaccination coverage target set by the World Health Organisation (WHO) had been met [1]. Vaccine uptake is driven by both accessibility and demand.

The evidence from low- and lower-middle- income countries shows that vaccine acceptance is linked to various demographic, socioeconomic and biomedical indicators. Studies have found that individuals who are older [2–9], high-income earners [4, 6, 10, 11], married [3, 10, 12, 13], employed [12, 13], and employed as healthcare workers [14, 15] or entrepreneurs [6, 7] are more likely to accept the vaccine. Females compared to males [3, 6, 10, 12–14, 16], and those employed as day labourers, agricultural workers or housewives [2, 17] compared to those with a salaried job are less likely to accept the vaccine. Though, some disagreement exists on the relevance of individual characteristics. Some studies showed older individuals [10, 12, 18] and high-income earners [17] to be less likely to accept the vaccine, and females [17] to be more likely to accept the vaccine. There is even less agreement between many studies on the association between educational attainment level or rural versus urban residency on vaccine acceptance [2–4, 6, 9, 10, 12, 13, 17–23]. Socioeconomic indicators have focused on income bracket, employment category, educational attainment, and urban versus rural residency, yet no studies have looked at ownership of resources such as land or formal identification, or government documents that enable lower-income families to access financial support and subsidised healthcare. No studies were found to have looked at vaccination among minority ethnic groups or those with inadequate household water, sanitation, and hygiene (WASH) practices. There also has been a focus on vaccine acceptance rather than vaccine uptake. It is important to study vaccine uptake as vaccine acceptance is not possible if one does not have access to a vaccine. Furthermore, high vaccine acceptance of 80.3% has been reported in low- and middle-income countries (LMICs) including India where it is highest (84%) [24].

In India, vaccination guidelines were based on individual age and comorbidities to reduce COVID-19 sequelae [25]. Previous studies have assessed the association between comorbidities and vaccine acceptance in low and lower-middle-income countries and found that those reporting a medical comorbidity were more likely to accept a vaccine [21]. Other studies have looked at factors associated with COVID-19 transmission and their relationship with vaccine acceptance. Knowing an individual with COVID-19 or reporting no prior SARS-CoV-2 infection were both negatively associated with vaccine acceptance [15, 17]. Receiving a negative SARS-CoV-2 test was positively associated with vaccine acceptance [4], and COVID-19 contact was positively associated with vaccine receipt [21]. These inconsistent findings may reflect different understandings of vaccines with regards to natural immunity and vaccine derived immunity.

We examined factors associated with the receipt of a COVID-19 vaccine and the timing to vaccine receipt for individuals in rural India. The aim of this study was to examine the relative importance of biomedical factors versus demographic and socioeconomic characteristics. We used cross-sectional data from a census of individuals residing in a rural village in India to answer the following question. What influences the likelihood that an individual receives at least one dose of a COVID-19 vaccine?

## Methods

### Vaccine policies in India

India is the second most populated country in the world [26]. It commenced its own vaccination programme on 16 January 2021 and shortly after, as it was a major vaccine manufacturer, exported vaccines internationally [27–29]. The second peak in COVID-19 infections and deaths in March 2021, followed by a spike in vaccine uptake, led to a shortfall in available vaccines and a behind-schedule vaccine distribution [27–29]. Vaccines became privately available on 1 May 2021 [29].

We assessed vaccine receipt from 21 June 2021 to 8 September 2022 during which time vaccine eligibility changed in India. During the time period of our study there were no reported vaccine shortages despite shortages being reported in March 2021. At the time our study commenced, all Indian residents over the age of 18 years were eligible for vaccination. Partway through our study, on 3 January 2022 and 14 March 2022, Indian residents aged 15 to 18 years and 12 to 14 years, respectively, became eligible for vaccination [30, 31].

Prior to our study, the Indian government had rolled-out vaccination to health and frontline workers (from 16 January 2021), residents over the age of 60 years and residents aged 45 to 60 years with a qualifying comorbidity (from 1 March 2021), residents over the age of 45 years (from 1 April 2021), and residents over the age of 18 years (from 1 May 2021) [32].

### Ethics

Ethical approval for the data collection was obtained from the National Institute for Research in Tuberculosis-Institutional Ethics Committee (NIRT-IEC) and the approval number was 2020–033 (NCT04813328). All participants gave written informed consent in a language they understood; adults consented on behalf of children.

### Sampling and participants

A census was conducted of a rural village, Pagalmedu, in the Tiruvallur district of Tamil Nadu in India. This area was selected as it is a COVID-19 hotspot. Details on the rolling recruitment of the study are available online [33]. Participants were compensated 200 Indian Rupees for their time. Participants were excluded from the study if aged less than 5 years, had received recent anthelminthic treatment or if unable to provide a venous blood sample. A survey of 350 households containing 1263 residents was undertaken. Of all households, 88 households (25.1%, n = 88/350) refused to participate. These 88 households contained 239 residents. Of the 262 included households, 249 participants (24.3%, n = 249/1024) refused to participate (n = 58/249), were not available for screening (n = 72/249), were not eligible for participation (n = 89/249), or were living in another location at the time of screening (n = 30/249). Of a total of 1263 residents, 775 participants took part in the study.

Each participant completed a face-to-face interview at one time-point between 21 June 2021 to 8 September 2022 with one of 16 trained interviewers who used DFcollect (a tablet-based mobile application that allows users to perform online and offline data collection for a DFdiscover study). All collected data were managed using DFexplore 2021 version 5.5.0. All participants underwent a clinical examination and provided a venous blood sample.

### Outcomes

The primary outcome was binary and defined as the receipt of at least one COVID-19 vaccine dose prior to the day of data collection. Self-reported vaccination receipt was validated using the national CoWIN digital application that held a record of every COVID-19 vaccination in India. The secondary outcome was binary and defined as the receipt of first COVID-19 vaccine dose within 30 days of eligibility compared to greater than 30 days or no vaccination.

### Demographic and socioeconomic variables

Individual-level demographic and socioeconomic data included binary variables: sex (female, male), ethnicity (minority, majority tribe) and marital status (not married, married). Minority tribe included participants from the Telugu (n = 147) or Urudhu (n = 1) tribes whilst the majority tribe included participants from the Tamil tribe (n = 502) only. The not married category included those who were separated (n = 3), widowed (n = 60), divorced (n = 1), and never married (n = 141). Categorical variables included age (years) (≤18, 19–44, 45–60, >60) and occupation (salaried, retired or unemployed, housewife, student, self-employed agricultural workers, self-employed non-agricultural workers, rural employment scheme). Education was an ordinal variable from 0–6 (no formal schooling, primary, high, secondary, senior secondary, university, post-graduate).

Household-level socioeconomic binary variables included ownership of the following household resources: land, electricity supply, permanent account number (PAN) card, ration card and Aadhaar card in comparison to no ownership, and usual use of private healthcare compared to government healthcare (n = 444), traditional healers (n = 2), or no healthcare (n = 2). Household WASH exposures were defined in line with the World Health Organisation (WHO)/UNICEF Joint Monitoring Programme [34]. Improved sanitation referred to the use of toilets that separated excreta from human contact. Protected water referred to safe drinking water. Improved hygiene referred to households observed to have a sink, running water, and soap.

### Biomedical variables

Self-reported biomedical variables focused on COVID-19 related care, travel, and illness. Any hospital admission within 12 months was coded as binary. Travel history was defined as local travel only versus any inter-city and inter-district travel. COVID-19 contact history was defined as no known contact compared to known contact with a confirmed or suspected case. It was noted whether a household was ever in a containment zone (never or unknown versus previously or currently). Binary indicators were constructed to assess if an individual had a COVID-19 test ever taken, received a positive COVID-19 PCR result (negative, unknown or not taken versus positive result), had any past (within three months) or current COVID-19 symptoms, and had any past (ever) or concomitant use of COVID-19 related medications. Lists of included COVID-19 symptoms and COVID-19 related medications are included in the Supplementary Methods in S1 File.

Clinical examinations were used to collect weight (kg), height (cm), neck circumference (cm), and vital signs (blood pressure (mmHg). Heart rate (HR) (beats per minute), respiratory rate (RR) (breaths per minute), oxygen saturations (Sp02%), and temperature (ºC)) were recorded and phlebotomy was undertaken for glycated haemoglobin (HbA1c) (%). Standard cut-offs were used to categorise BMI, HbA1c, HTN, HR and RR for adults and children (Supplementary Methods in S1 File).

Participants self-reported past or current medical conditions under 17 anatomical sites. This was used to create two exposures. The first was a binary variable indicating if the participant had one of twenty conditions specified by the Indian government as enabling vaccine priority and this included conditions such as moderate or severe valvular heart disease (conditions listed in S2 Table in S1 File). To enable comparisons with other published studies, a second comorbidity variable was created for use in a sensitivity analyses. This second comorbidity variable was a binary variable indicating if the patient had any current medical condition compared to past or no conditions. All self-reported medical conditions, including if they are past or current, are listed in the S3 Table in S1 File.

### Statistical analysis

Stata version 17.0 was used for the statistical analysis. A breakdown of the derivation of the final number of participants analysed is as follows. Of 775 surveyed participants, 450 had been vaccinated with 95.3% (n = 429/450) following vaccine eligibility criteria based on age and comorbidities. Those who did not follow vaccine eligibility criteria (4.7%, n = 21/450) were within the following age bands: 12–14 (n = 3), 15–18 (n = 5), 19–44 (n = 7) and 45–60 (n = 6). Participants ineligible for the COVID-19 vaccine were excluded. Of the 775 surveyed participants, 659 were eligible for COVID-19 vaccination. Those who were ineligible for COVID-19 vaccination (n = 116), and excluded from the study, included those aged 15–18 and 12–14 years with study entry on or before 3 January 2022 and 14 March 2022 respectively. Using complete case analysis, 650 participants were included. Thus, 1.4% (n = 9/659) of participants were excluded due to missing data on BMI (n = 1), HbA1c (n = 4), HTN (n = 4), HR (n = 3) and RR (n = 2). Some participants had data missing across multiple variables. Four participants with missing data on BMI were kept in the study as their neck circumference was used to impute a BMI category: normal weight (n = 1), overweight (n = 2) and obese (n = 1) (S4 Table in S1 File).

Logistic regressions were used for the binary outcomes of vaccine receipt and timing to vaccine receipt. Across all models, variables were excluded from regression models if there were <5 participants per strata due to lack of variation and power for analysis. For each model, all variables were tested using likelihood ratio (LR) tests to compare a univariable logistic regression model to an empty model. Variables were selected for the multivariable logistic regression model if the LR chi-squared p-value was <0.10. A 10% significance level was chosen to maximise power due to study sample size.

For the main model of vaccine receipt, 650 eligible participants were included. Two sensitivity analyses were conducted. The first sensitivity analysis was conducted to look at vaccine receipt amongst all surveyed participants (both eligible and ineligible) without any missing data (n = 743). This was conducted as 21 participants received a vaccine earlier than recommended by national guidelines. Of these 21 participants, 13 were aged over 18 years old and may have been vaccinated due to being a frontline worker, which was data we did not collect. The second sensitivity analysis was almost identical to the main model of vaccine receipt (n = 650), but the medical comorbidity variable was recoded: instead of looking at participants with and without an Indian-specific medical condition that enabled vaccine priority, participants were classified as to whether they had a current medical condition versus a past or no medical condition. For the main model of timing to vaccine receipt, 647 participants were included. Three participants were excluded from this model due to a missing date of COVID-19 vaccination. No additional sensitivity analyses were undertaken.

Model robustness checks included the following. Multicollinearity between independent variables was determined using a variance inflation factor (VIF) cut-off of 10 and based upon this, no collinear variables were removed from fully adjusted models [35]. Independent variables were either binary or categorical thus no outliers were removed from the dataset. Model prediction was assessed using 5-fold cross-validation to provide the area under the receiver operating characteristic curve [36]. In multivariable models, clustered (robust) standard errors were used to account for household clustering of outcomes due to the sampling of all individuals within a household [37]. Our study was cross-sectional in design, and participants were not followed-up to observe if they were vaccinated, so the day of data collection was included in all the statistical models as a confounding factor.

### Synthesis of results

The Health Belief Model (HBM) is a behaviour change model that is commonly used to predict and explain the uptake of health services [38]. To synthesise our results, we used this model to show the key determinants of vaccine receipt compared to timing to vaccine receipt. The determinants were grouped under two categories: susceptibility and severity to infection, and benefits and barriers to vaccination, to explain how they affected the main outcomes.

## Results

Overall, 68.0% (n = 442/650) of vaccine eligible participants received one vaccine dose. Of these, 49.3% (n = 218/442) and 50.0% (n = 221/442) had scheduled and received their second dose with 0.7% (n = 3/442) unsure. One-third were fully vaccinated (34.0%, n = 221/650). Most participants had received CoviShield (93.0%, n = 411/442) compared to Covaxin (2.5%, n = 11/442) or unknown (0.5%, n = 2/442). No vaccine refusals were reported.

**Fig 1:**
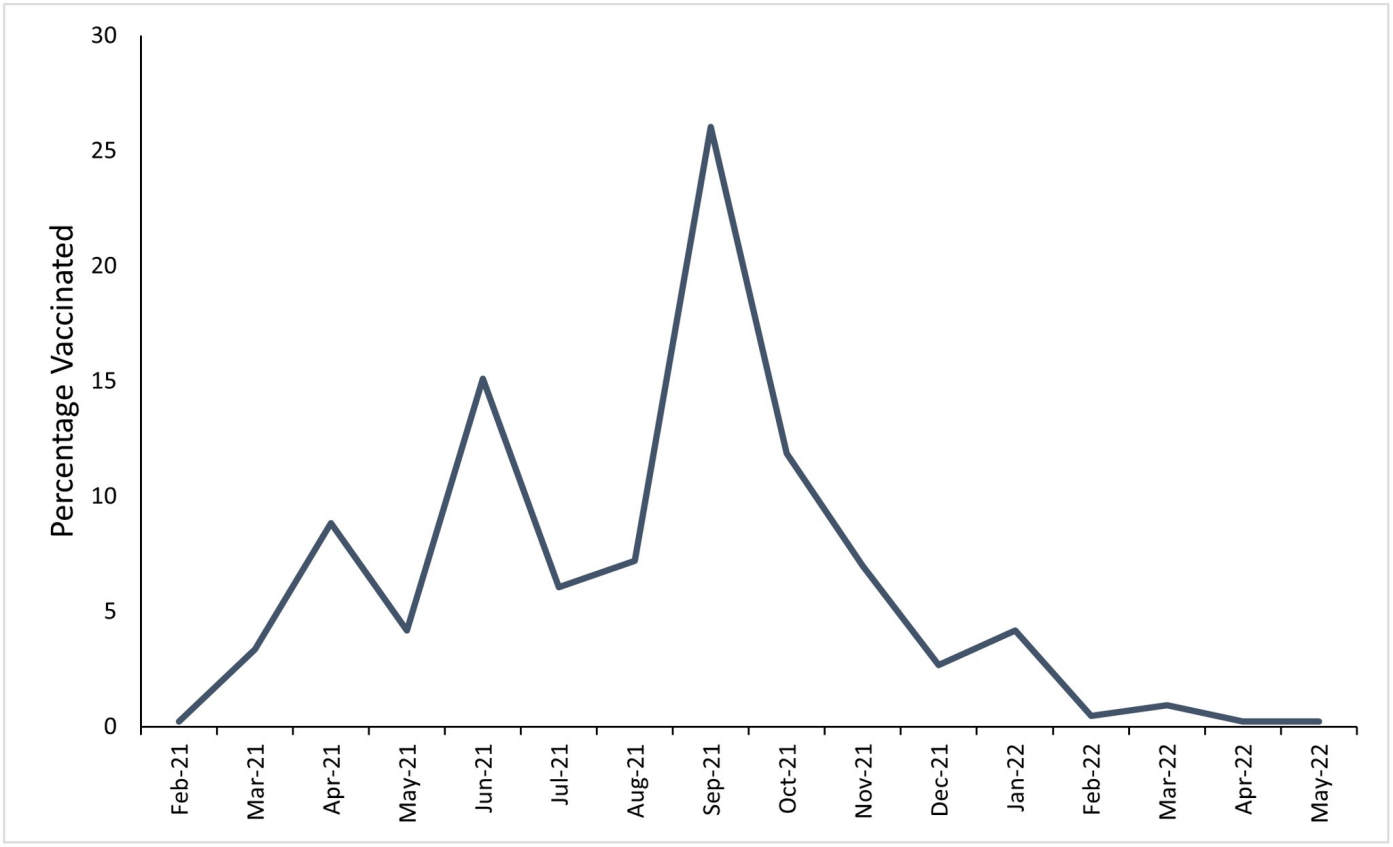
Percentage of vaccines given per month of data collection among vaccinated participants.

Peaks in vaccination occurred in June and September 2021 (Fig 1). The proportion of patients vaccinated per month of data collection increased over the study period from 46.9% in June 2021 to 68.0% in September 2022. The median day of data collection for unvaccinated participants was 74 (IQR: 44–232) compared to 211.5 (IQR: 130–373) for vaccinated participants.

**Fig 2:**
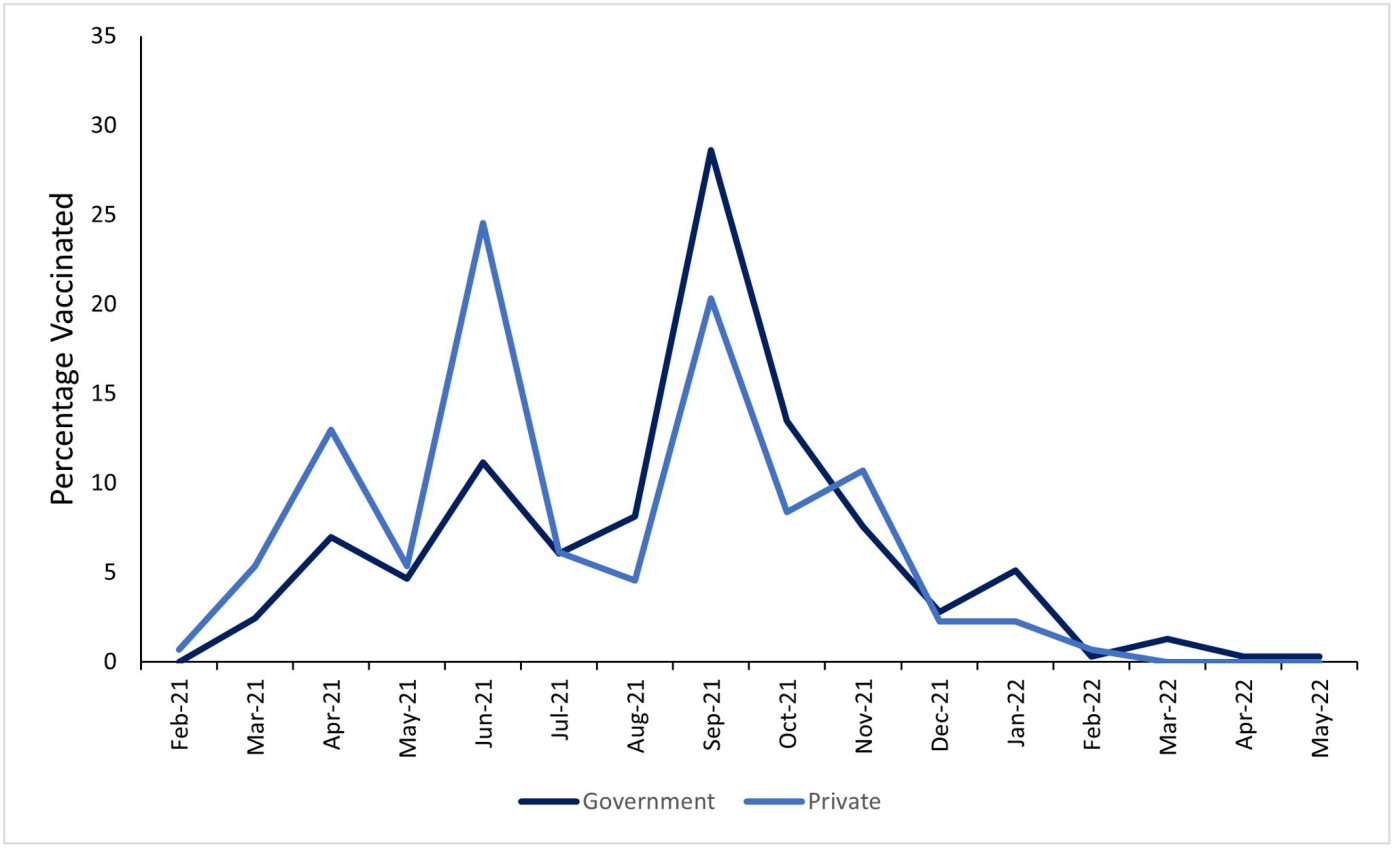
Percentage of vaccines given per month of data collection split by healthcare use. The percentage was calculated among only those offered at least one vaccine dose.

The median number of days to first vaccination was 123 (IQR: 44–175). Of those vaccinated with a known vaccination date, 17.1% (n = 75/439) were vaccinated within 30 days of eligibility. Of those who received a vaccine prior to their eligibility date, 42.9% (n = 9/21) used private healthcare. The peak in vaccinations for those households reporting typical use of private healthcare was June 2021 compared to September 2021 in for households reporting that they generally use government healthcare providers (Fig 2).

### Participant characteristics

Participant demographic, SES and WASH characteristics are displayed in Table 1. The majority of participants were aged 19–44 (55.4%) and evenly split by gender (female 52.9%). Almost one-sixth of participants had no formal schooling (14.6%). Most individuals were employed (64.2%, n = 417/650) with the majority of these in self-employed work (65.5%, n = 236/417). Just over one-third of individuals belonged to households that typically used private healthcare. Over 68% of participants were in a household where a member had a PAN card.

**Table 1:** Participant demographic, SES and WASH characteristics by vaccination status.

Determinants	Total	Unvaccinated	Vaccinated	
	n (%)	n (%)	n (%)	
	N = 650	N = 442	N = 208	Chi^2^ p-value
**Demographic**				
Age category (years)				
≤18	34 (5.2)	12 (5.8)	22 (5.0)	0.77
19–44	360 (55.4)	109 (52.4)	251 (56.8)	
45–60	196 (30.2)	67 (32.2)	129 (29.2)	
>60	60 (9.2)	20 (9.6)	40 (9.0)	
Male	306 (47.1)	93 (44.7)	213 (48.2)	0.41
Majority tribe (Tamil)	502 (77.2)	157 (75.5)	345 (78.1)	0.47
Married	445 (68.5)	154 (74.0)	291 (65.8)	0.04
Educational attainment				
No formal schooling	95 (14.6)	25 (12.0)	70 (15.8)	<0.01
Primary	65 (10.0)	30 (14.4)	35 (7.9)	
High	134 (20.6)	64 (30.8)	70 (15.8)	
Secondary	129 (19.8)	36 (17.3)	93 (21.0)	
Senior Secondary	69 (10.6)	17 (8.2)	52 (11.8)	
University	132 (20.3)	29 (13.9)	103 (23.3)	
Post-graduate	26 (4.0)	7 (3.4)	19 (4.3)	
Occupation group				
Salaried	144 (22.2)	24 (11.5)	120 (27.1)	<0.01
Retired or unemployed	49 (7.5)	18 (8.7)	31 (7.0)	
Housewife	132 (20.3)	64 (30.8)	68 (15.4)	
Student	52 (8.0)	12 (5.8)	40 (9.0)	
Self-employed agricultural	100 (15.4)	27 (13.0)	73 (16.5)	
Self-employed non-agricultural	136 (20.9)	59 (28.4)	77 (17.4)	
Rural Employment Scheme	37 (5.7)	4 (1.9)	33 (7.5)	
**SES**				
Private healthcare use	202 (31.1)	73 (35.1)	129 (29.2)	0.13
Household land ownership	582 (89.5)	164 (78.8)	418 (94.6)	<0.01
Household electricity supply	648 (99.7)	207 (99.5)	441 (99.8)	
Household Aadhaar card ownership	650 (100)	208 (100)	442 (100)	
Household PAN card ownership	444 (68.3)	124 (59.6)	320 (72.4)	<0.01
Household ration card ownership	618 (95.1)	189 (90.9)	429 (97.1)	<0.01
**WASH**				
Improved Sanitation	528 (81.2)	187 (89.9)	341 (77.1)	<0.01
Protected Water source	647 (99.5)	207 (99.5)	440 (99.5)	
Improved Hygiene	623 (95.8)	196 (94.2)	427 (96.6)	0.16

SES = socioeconomic status; WASH = water, sanitation, and hygiene.

Participant biomedical characteristics are displayed in Table 2. A minority of participants reported a medical condition prioritised by the Indian government (3.2%). Of those individuals aged over 60 years, 15% (n = 9/60) had a priority medical condition compared to 0% (n = 0/34) aged ≤18 years; 0.6% (n = 2/360) aged 19–44 years and 5.1% (n = 10/196) aged 45–60 years. Two-thirds of participants were overweight or obese. One-fifth of individuals had a raised HbA1c and of these, 53.7% self-reported diabetes mellitus. Nearly 25% of participants had HTN diagnosed through clinical examination yet only 24.7% (n = 38/154) of those individuals who were diagnosed self-reported HTN. For people with an abnormal HR, 86.0% (n = 49/57) were tachycardic and 14.0% (n = 8/57) were bradycardic. The median HR for tachycardic participants was 107bpm (IQR: 104-111bpm) and for bradycardic participants was 54bpm (IQR: 51–58). Very few individuals were diagnosed with abnormal RR (1.2%, n = 8/650) or abnormal SpO2 (0.2%, n = 1/650). No participants had a fever at the time of survey. Only 1.2% of participants reported a hospital admission within 12 months.

**Table 2:** Participant biomedical characteristics by vaccination status.

Determinants	Total	Unvaccinated	Vaccinated	
	n (%)	n (%)	n (%)	
	N = 650	N = 442	N = 208	Chi^2^ p-value
Recent hospital admission	8 (1.2)	6 (2.9)	2 (0.5)	<0.01
Priority medical condition	21 (3.2)	12 (5.8)	9 (2.0)	0.01
BMI Category				0.04
Normal weight	169 (26.0)	55 (26.4)	114 (25.8)	
Underweight	52 (8.0)	18 (8.7)	34 (7.7)	
Overweight	122 (18.8)	26 (12.5)	96 (21.7)	
Obese	307 (47.2)	109 (52.4)	198 (44.8)	
Raised HbA1c	136 (20.9)	52 (25.0)	84 (19.0)	0.08
Hypertension	154 (23.7)	54 (26.0)	100 (22.6)	0.35
Abnormal heart rate	57 (8.8)	24 (11.5)	33 (7.5)	0.09
Abnormal respiratory rate	8 (1.2)	4 (1.9)	4 (0.9)	0.27
Fever	0 (0)	0 (0)	0 (0)	
Low oxygen saturations	1 (0.2)	0 (0)	1 (0.2)	
Travel History	442 (68.0)	111 (53.4)	331 (74.9)	<0.01
COVID-19 contact	25 (3.8)	14 (6.7)	11 (2.5)	<0.01
Household in containment zone	5 (0.8)	2 (1.0)	3 (0.7)	
COVID-19 test taken	141 (21.7)	31 (14.9)	110 (24.9)	<0.01
Positive COVID-19 PCR result	12 (1.8)	7 (3.4)	5 (1.1)	0.05
COVID-19 symptoms	45 (6.9)	19 (9.1)	26 (5.9)	0.13
COVID-19 medications taken	17 (2.6)	10 (4.8)	7 (1.6)	0.02

BMI = body mass index, HbA1c = glycated haemoglobin, PCR = polymerase chain reaction

Despite most participants having travelled into another city or district (68.0%), only 3.8% of participants reported known or suspected contact with COVID-19. Similarly, only 6.9% and 1.8% of individuals, respectively reported experiencing of COVID-19 symptoms or receiving a positive PCR test result. Less than 1% of individuals belonged to a household that was ever located within a containment zone.

### Determinants of vaccine receipt and vaccine timing

**Fig 3:**
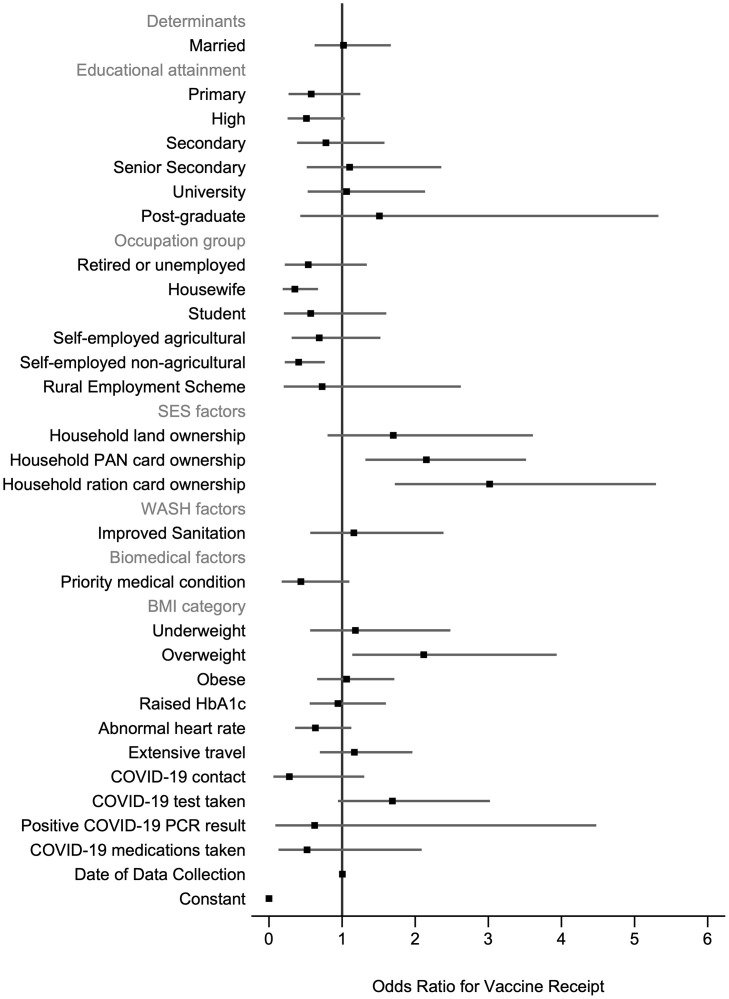
Determinants of vaccine receipt. N = 650 participants. 5-fold cross-validated mean area under the receiver operating curve = 0.737 (SD 0.030). Robust standard errors are clustered at the household level. There are 262 clusters. BMI = body mass index, HbA1c = glycated haemoglobin, PAN = permanent account number; PCR = polymerase chain reaction; SES = socioeconomic status; WASH = water, sanitation, and hygiene.

For the model examining vaccine receipt, all crude odds ratios (ORs) and univariable LR test results are given in the S5 Table in S1 File. Seventeen variables were selected for inclusion in the fully adjusted model (Fig 3, S6 Table in S1 File). Of these variables, occupation group, household PAN card ownership and household ration card ownership were significantly associated with odds of vaccination. Participants who worked as housewives (adjusted odds ratio (AOR) = 0.35; 95% CI: 0.19–0.67) or self-employed non-agricultural workers(AOR = 0.41; 95% CI: 0.22–0.76) compared to salaried workers were less likely to be vaccinated. Participants who owned a PAN card (AOR = 2.15; 95% CI: 1.32–3.52) or a ration card (AOR = 3.02; 95% CI: 1.72–5.29) compared to no ownership were between 2–3 times more likely to receive a vaccine. Although BMI was not significant across all categories (group p-value>0.05), participants who were overweight compared to a normal weight were significantly more likely to be vaccinated (AOR = 2.12; 95% CI: 1.14–3.94).

**Fig 4:**
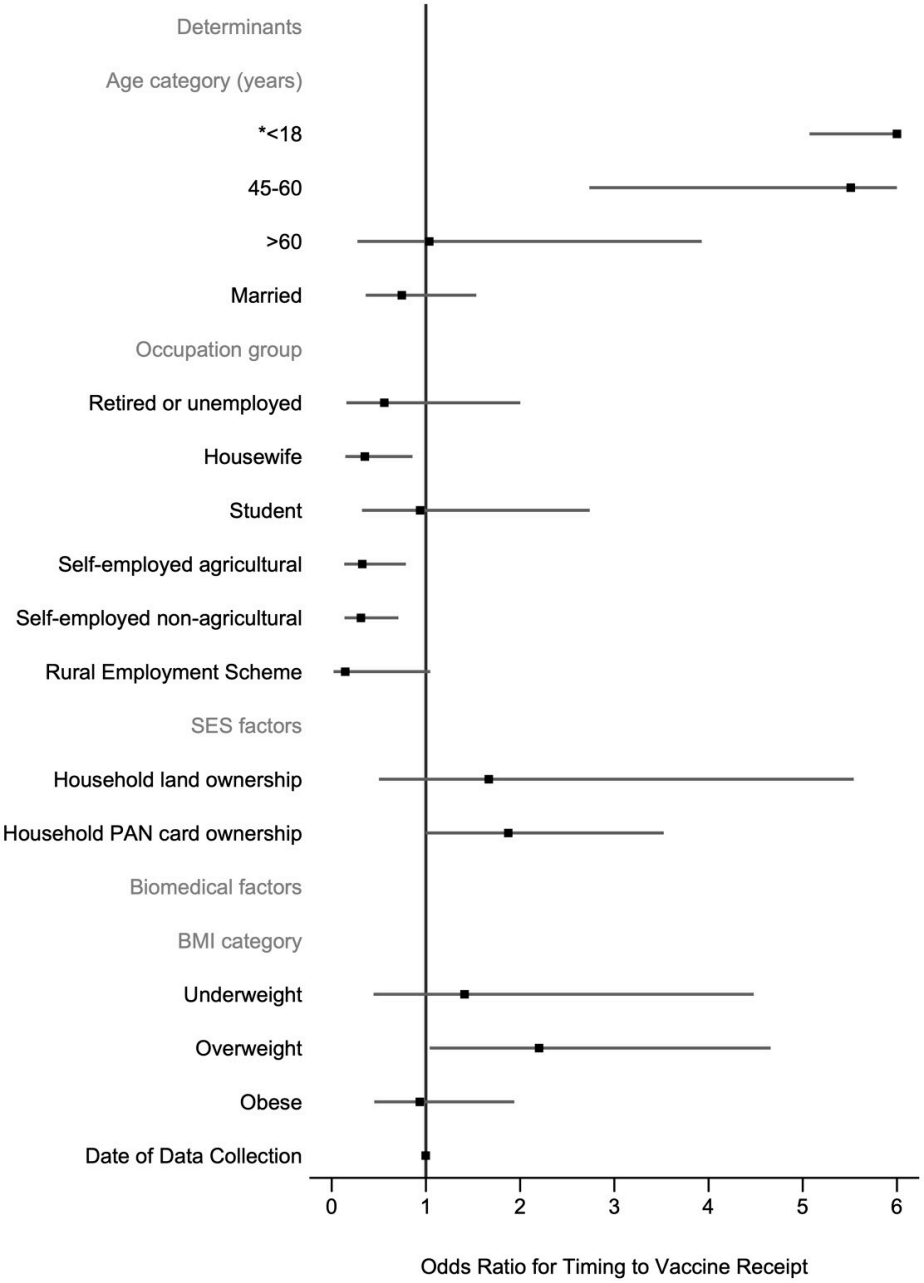
Determinants of the timing to vaccine receipt. N = 647 participants. 5-fold cross-validated mean area under the receiver operating curve = 0.663 (SD:0.082). Robust standard errors are clustered at the household level. There are 262 clusters. BMI = body mass index; PAN = permanent account number; SES = socioeconomic status.

For vaccine receipt within 30 days of eligibility, all crude ORs and univariable LR test results are given in the S7 Table in S1 File. Seven variables were selected for inclusion in the fully adjusted model (Fig 4, S8 Table in S1 File). Of these variables, age, occupation group, household PAN card ownership and BMI category were significantly associated with odds of timing to vaccine receipt. Those aged ≤18 years (AOR = 17.74; 95% CI: 5.07–62.03) and aged 45–60 (AOR = 5.51; 95% CI: 2.74–11.10) were more likely to receive a vaccine within 30 days of eligibility compared to those aged 19–44 years. Participants who worked as housewives (AOR = 0.35; 95% CI: 0.14–0.86), self-employed agricultural workers (AOR = 0.32; 95% CI: 0.13–0.79) or self-employed non-agricultural workers (AOR = 0.31; 95% CI: 0.14–0.71) compared to salaried workers were less likely to receive a vaccine within 30 days of eligibility. Participants who owned a PAN card (AOR = 1.87; 95% CI: 1.00–3.52) were more likely to receive a vaccine within 30 days of eligibility however, this was borderline significant. In contrast to vaccine receipt, BMI categories were significant overall (group p-value<0.05). Participants who were overweight were significantly more likely to be vaccinated within 30 days of eligibility (AOR = 2.20; 95% CI: 1.04–4.66).

### Sensitivity analyses

**Fig 5:**
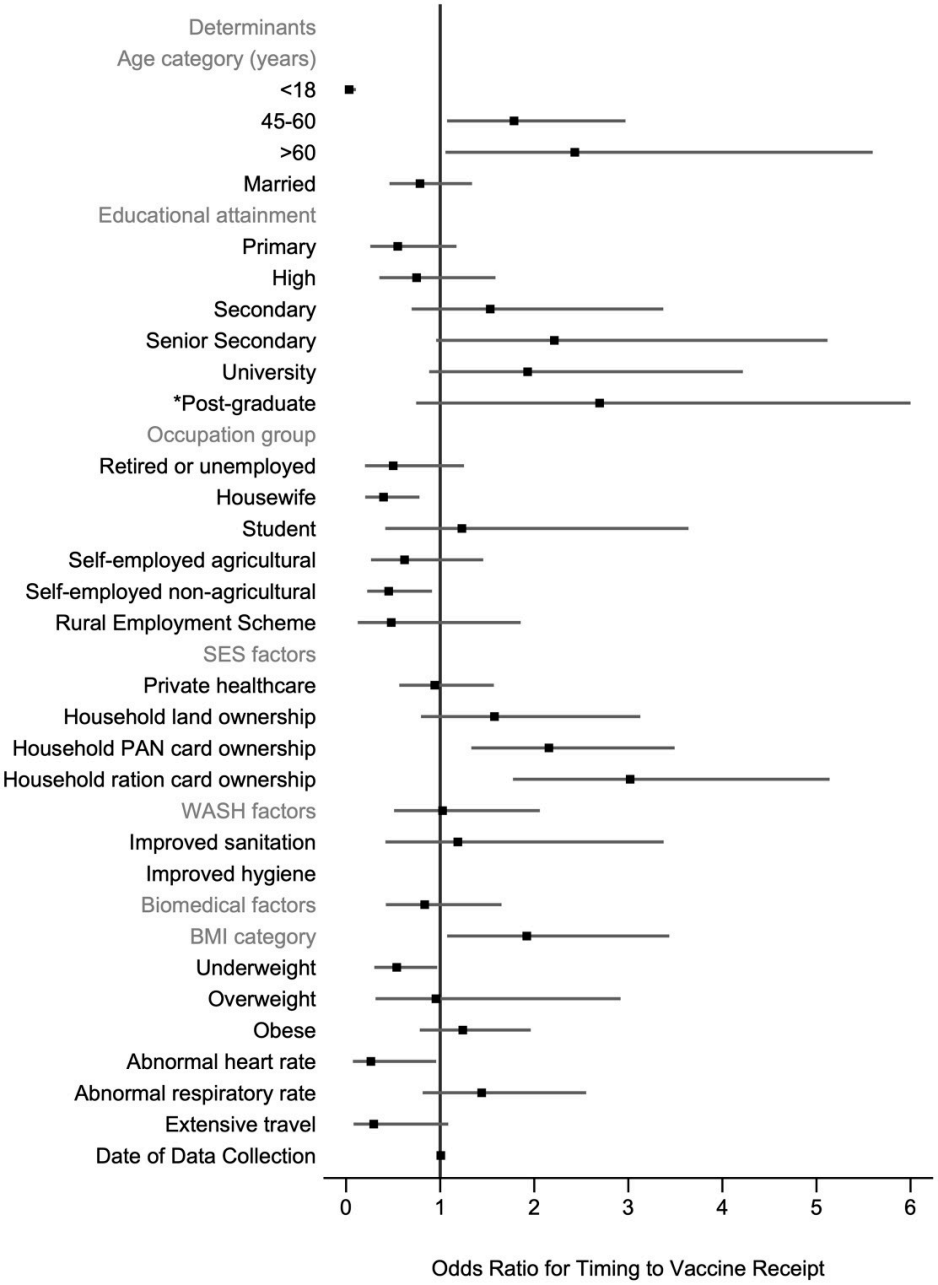
Determinants of vaccine receipt in all surveyed participants. N = 743 participants. 5-fold cross-validated mean area under the receiver operating curve = 0.802 (SD: 0.046). Robust standard errors are clustered at the household level. There are 262 clusters. BMI = body mass index; PAN = permanent account number; SES = socioeconomic status; WASH = water, sanitation, and hygiene. *95% upper limit confidence interval for post-graduate educational attainment is 9.66.

Differences were found between vaccine eligible and ineligible participants related to the age of ineligible participants (all ≤18 years) (S9 Table in S1 File). We compared the findings from the main model of our primary outcome (n = 650) to a model using all surveyed participants and not excluding vaccine ineligible participants (n = 743) to look at the primary outcome. Due to a larger sample size in this model, additional variables (age category, private healthcare use, improved hygiene, abnormal HR, abnormal RR and COVID-19 symptoms) were selected from univariable LR tests (S10 Table in S1 File) for inclusion in the fully adjusted model (Fig 5, S11 Table in S1 File). Three variables were not excluded that had been selected in our main model (priority medical condition, raised HbA1c and positive COVID-19 PCR result). In the model using all surveyed participants (n = 743), occupation group, household PAN card ownership, household ration card ownership and being overweight all remained significantly associated with the odds of vaccine receipt and the coefficients were very similar to the main model which had excluded vaccine ineligible participants (n = 650). History of COVID-19 contact was borderline significant in the model using all surveyed participants and was associated with a lower odds of vaccine receipt (AOR = 0.27; 95% CI: 0.07–0.98).

**Fig 6:**
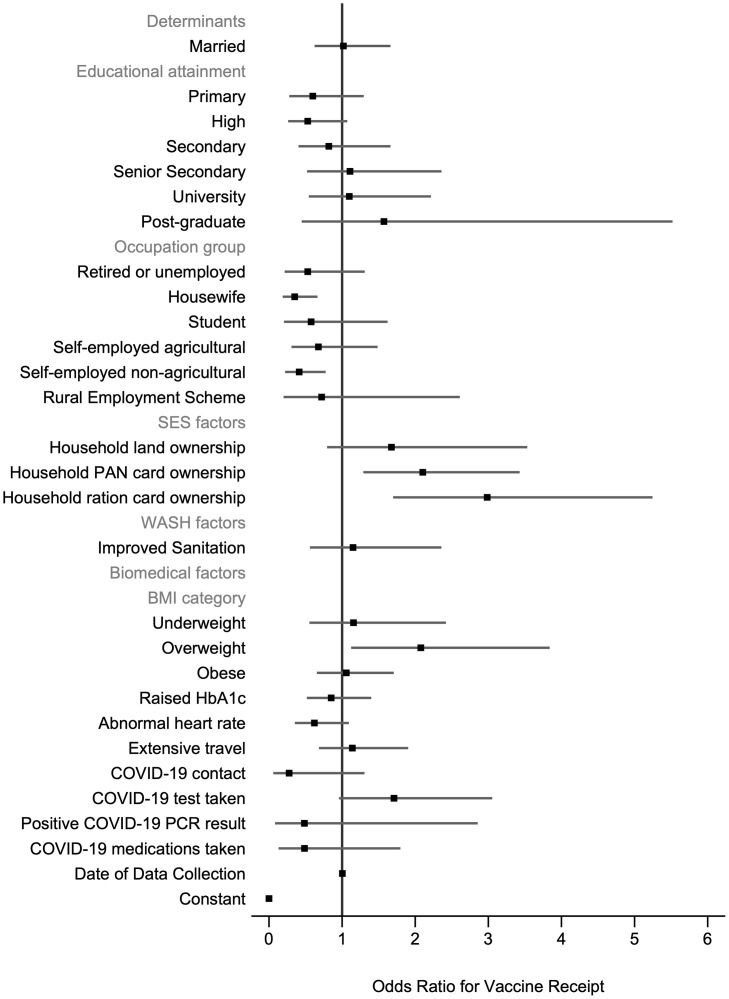
Determinants of vaccine receipt using current medical condition. N = 650 participants. 5-fold cross-validated mean area under the receiver operating curve = 0.738 (SD:0.032). Robust standard errors are clustered at the household level. There are 262 clusters. BMI = body mass index, HbA1c = glycated haemoglobin, PAN = permanent account number; PCR = polymerase chain reaction; SES = socioeconomic status; WASH = water, sanitation, and hygiene.

In our main model for COVID-19 vaccine receipt, the variable indicating a medical condition enabling vaccine priority was selected for inclusion but was not significant after multiple adjustment. When this variable was replaced with the variable indicating any current medical condition (versus past or none), the unadjusted association was insignificant (crude OR = 0.96; 95% CI:0.59–1.54) (S12 Table in S1 File) and the variable was not entered into the multivariable regression model (Fig 6, S13 Table in S1 File). The proportion of participants who self-reported a current medical condition differed by age-bracket: ≤18 years (0%, n = 0/34), 19–44 years (10.0%, n = 36/360), 45–60 years (26%, n = 51/196) and >60 years (36.7%, n = 22/60).

Household-level variables were removed from the model of vaccine receipt to assess confounding and proxy indicators. When both PAN and ration card ownership were removed from the model, the association between household land ownership and vaccine receipt became significant (AOR = 2.33; 95% CI: 1.07–5.06). The association between occupation and vaccine receipt remained unchanged and no other variables became significant. When PAN card ownership only was removed from the model, the association between household land ownership and vaccine receipt became borderline insignificant (AOR = 1.95; 95% CI: 0.90–4.25) and the magnitude of the association between ration card ownership and vaccine receipt remained similar (AOR = 3.23; 95% CI: 1.93–5.41). When ration card ownership only was removed from the model, the association between household land ownership and vaccine receipt became borderline insignificant (AOR = 2.02; 95% CI: 0.93–4.41) and the magnitude of the association between PAN card ownership and vaccine receipt remained similar (AOR = 2.18; 95% CI: 1.34–3.56).

**Fig 7:**
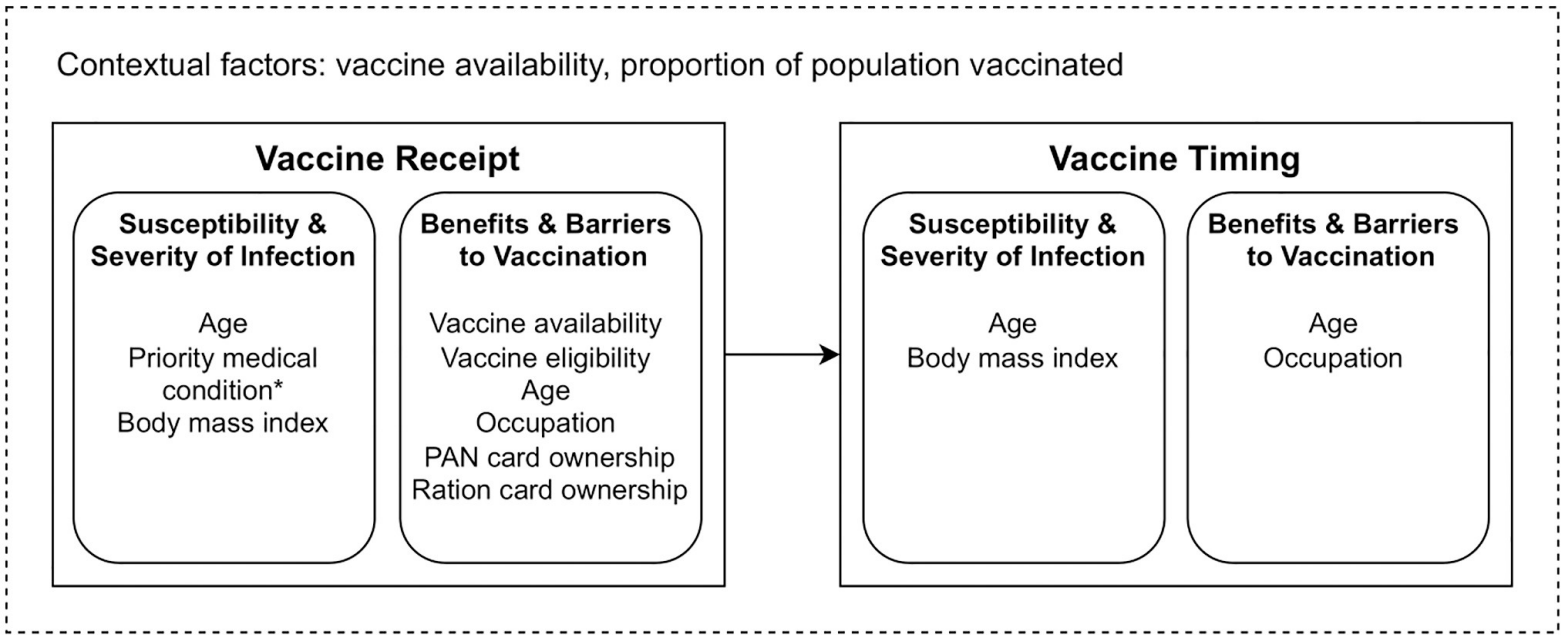
Health belief model of vaccine receipt and timing to vaccine receipt. *The association between priority medical condition and vaccine receipt was borderline significant. PAN = permanent account number.

A conceptual framework summarising the results from the vaccine receipt and vaccine timing model are presented in Fig 7. These factors collectively determined if an individual took up an available vaccine. As a higher proportion of the population get vaccinated, we assumed that timing to vaccine receipt was reduced as individuals may be able to make a quicker assessment of vaccine uptake.

## Discussion

Many low and lower-middle- income countries did not meet the vaccination coverage targets set by COVAX and the WHO [1]. In this study, we used cross-sectional data from a rural village census of 262 households in India to examine demographic, socioeconomic, and biomedical factors associated with vaccine receipt and timing to vaccine receipt. Our main findings were that demographic and socioeconomic factors, including ownership of formal government identification, rather than COVID-19 specific conditions or comorbidities were linked to vaccine receipt and timing of receipt.

Our results are consistent with similar studies undertaken in Tamil Nadu, India that looked at factors associated with vaccine acceptance. Two community-based cross-sectional studies conducted in January 2021 and March to May 2021 found that socio-demographic characteristics including higher education and being employed or of a higher-income employment class were significantly associated with vaccine acceptance [39, 40]. One study found that older individuals [39] were more likely to accept a vaccine whilst the other study found that younger individuals were more likely to show vaccine acceptance [40]. Unlike our study, it was found that women [39], married individuals [40] and rural residents [39] were less likely to accept a vaccine. Regarding biomedical factors, we found a borderline significant association between self-reported priority medical condition and vaccine receipt but no association between current medical condition and vaccine receipt. Likewise, a similar study found no association between self-reported physician confirmed illness and vaccine acceptance [40]. A third Indian study found that socioeconomic deprivation was negatively associated with vaccine coverage [41].

From univariable models, the variables and number of variables that were selected for models examining vaccine receipt and timing to vaccine receipt differed. This suggests that different factors may explain vaccine receipt compared to timing to vaccine receipt—educational attainment, household ownership of a ration card, improved household sanitation facilities and individual biomedical factors were linked to vaccine receipt whilst age was more predictive of timing to vaccine receipt.

We have developed a conceptual framework, adapted from the HBM, that can help to explain our main findings. This model shows how various determinants are linked to vaccine receipt and timing to vaccine receipt. Diagnosis of a priority medical condition or a higher BMI can lead an individual to feel more susceptible to a more severe COVID-19 infection. Although the association between priority medical condition and vaccine receipt was borderline significant, we did see that those aged 45–60 years were more likely to receive a vaccine within 30 days of eligibility compared to those aged 19–44 years and this may have been due to a higher prevalence of priority medical conditions (5.1% vs 0.6%) or current medical conditions (26% vs 10%) in this older age group. As this variable was self-reported, it is possible that an association was missed due to under-reporting. Other factors including vaccine eligibility, age, occupation, household ownership of a PAN card and household ownership of a ration card can affect the benefits and barriers to COVID-19 vaccination at an individual level. Our study looked at timing to vaccine receipt as an outcome and our model takes into account how occupation and age affected timing to vaccine receipt. As a higher proportion of the population became vaccinated, we saw a decrease in timing to vaccine receipt as demonstrated by our finding that the youngest age category (eligible for vaccination at a later time period) were most likely to be vaccinated within 30 days of eligibility.

Household ownership of PAN or ration cards were both positively correlated to vaccine receipt, despite the PAN and ration cards representing high and low SES, respectively. A PAN card is a prerequisite for a salaried job in which one pays income tax. In India, ration cards are given to households as a form of household identification and to enable households living below the poverty line to buy subsidised grain under the public distribution system [42]. Previous studies have shown an association between higher income and vaccination [4, 6, 10, 11]. Other explanations for the association between possession of formal government identification and increased odds of vaccination include the possibility of these variables approximating land ownership, existence of vaccine mandates, improved vaccine access linked to a salaried job, or vaccine campaigns delivered as part of health promotion at ration centres. When both PAN and ration card were removed from the main model, household land ownership became significantly associated with vaccine receipt. In Tamil Nadu, vaccine mandates were introduced for access to workplaces [43] and public places [44, 45] thus increasing the perceived benefits to vaccination. Although these vaccine mandates were banned in May 2022, they may have positively influenced vaccine uptake beyond this time due to vaccine receipt becoming the norm [46]. Improved vaccine access linked to a salaried job could be due to vaccine access on the journey to or at work or through positive co-worker influence. As participants with formal government identification, such as PAN and ration cards, were significantly more likely to receive a vaccine, other studies should consider collecting this data when examining determinants of vaccine receipt. Further research might assess whether government interventions to increase vaccine uptake could be linked to ownership of formal government identification.

Housewives were less likely to be vaccinated and less likely to receive a vaccine within 30 days of eligibility. This could be due to gender, caste or SES, vaccine hesitancy, or selection bias. In this study, all housewives were female. Previous studies have shown that females are less likely to be vaccinated in India due to patriarchal sociocultural norms, poorer access to healthcare, concerns about effects on reproductive health, and misinformation linked to digital access [41, 47, 48]. However, we found no significant association between sex and odds of vaccination, and no collinearity between sex and occupation. Housewives might be of a lower caste or SES which in turn may correlate to reduce vaccine acceptance. Elsewhere housewives have been shown to have higher vaccine hesitancy [2]. In our study, we did measure self-reported vaccine refusals (N = 0), but this may have been underreported due to social desirability bias and the hesitancy to report negative outcomes. Alternatively, those who refused the vaccine might have been less likely to participate in our study.

Self-employed non-agricultural workers had a lower odds of vaccine receipt and both self-employed non-agricultural and agricultural workers were less likely to receive a vaccine within the first 30 days of eligibility. At a national level, agricultural work has declined in India since 2004 due to mechanisation of labour, with an increase in non-agricultural work [49]. Those working in self-employed jobs in rural India are often completing out-sourced informal work with low pay and high job precarity [49]. High proportions of self-employed non-agricultural (26.3%) and agricultural (35.5%) workers live in poverty [50]. Thus, our findings are consistent with previous research linking low income to reduced vaccination coverage [4, 6, 10, 11].

Age was linked to timing of vaccine receipt with those aged ≤18 years and 45–60 years significantly more likely to receive a vaccine within 30 days of eligibility compared to adults aged 19–44 years. Children may have been more likely to receive an early vaccine due to higher parental confidence in vaccine safety, and decreased perceptions of barriers to vaccination, due to the vaccine programme being established for 12 months.

This study used a comprehensive set of demographic, socioeconomic and biomedical variables to identify factors associated with vaccine uptake and timing of vaccine receipt in a rural population in India. We expect these findings to be generalisable to other rural villages in the Southern states of India with similar levels of socioeconomic status, disparities and access to health care—factors that differ widely between Southern and Northern India. The strengths of the study include the use of a census to survey an entire village, validation of the primary outcome of vaccination using the CoWIN application, and clinical examination to mitigate bias in self-reported health status. The main limitation of this study is that it was cross-sectional thus we were unable to determine if unvaccinated groups remained unvaccinated. There was an overall low prevalence of priority medical conditions thus we cannot rule out that this factor was not linked to vaccine uptake. Further studies should use linkage with medical records to determine if medical comorbidities, especially those linked to increased severity of COVID-19, are associated with vaccine uptake.

The main findings of our study present associations between possession of formal government identification, occupation, and age with vaccine receipt and timing to vaccine receipt. The findings of our study, if replicated longitudinally and in other settings, may be used to target groups for improved vaccine uptake in rural populations and future roll-out of COVID-19 precautionary (booster) doses.

## Supporting information

S1 File Supplementary Methods: List of included COVID-19 symptoms and COVID-19 related medications; S1 Table: Age-specific blood pressure cut-off values used to define HTN in children; S2 Table: List of medical conditions specified by the Indian government as enabling vaccine priority; S3 Table: Self-reported medical conditions listed under seventeen anatomical sites; S4 Table: Sex-specific neck circumference cut-off values used to categorise adults into BMI categories; S5 Table: Determinants of vaccine receipt; S6 Table: Determinants of timing to vaccine receipt; S7 Table: Participant characteristics split by vaccine eligibility; S8 Table: Determinants of vaccine receipt in all surveyed participants; S9 Table: Determinants of vaccine receipt using current medical condition; S10 Table: Determinants of vaccine receipt in all surveyed participants (univariable regression model); S11 Table: Determinants of vaccine receipt in all surveyed participants (multivariable regression model); S12 Table: Determinants of vaccine receipt using current medical condition (univariable regression model); S13 Table: Determinants of vaccine receipt using current medical condition (multivariable regression model).
(DOCX)

S2 File STROBE checklist. (DOC)
