# Supplementary material for: Relative contribution of biomedical, demographic, and socioeconomic factors to COVID-19 vaccine receipt in rural India
Source: PLoS One. 2024 Jun 24;19(6):e0305819. doi: 10.1371/journal.pone.0305819 (PMC11195973; doi:10.1371/journal.pone.0305819)
Supplement: S1 File — Supplementary Methods: List of included COVID-19 symptoms and COVID-19 related medications; S1 Table: Age-specific blood pressure cut-off values used to define HTN in children; S2 Table: List of medical conditions specified by the Indian government as enabling vaccine priority; S3 Table: Self-reported medical conditions listed under seventeen anatomical sites; S4 Table: Sex-specific neck circumference cut-off values used to categorise adults into BMI categories; S5 Table: Determinants of vaccine receipt; S6 Table: Determinants of timing to vaccine receipt; S7 Table: Participant characteristics split by vaccine eligibility; S8 Table: Determinants of vaccine receipt in all surveyed participants; S9 Table: Determinants of vaccine receipt using current medical condition; S10 Table: Determinants of vaccine receipt in all surveyed participants (univariable regression model); S11 Table: Determinants of vaccine receipt in all surveyed participants (multivariable regression model); S12 Table: Determinants of vaccine receipt using current medical condition (univariable regression model); S13 Table: Determinants of vaccine receipt using current medical condition (multivariable regression model). (DOCX) [file pone.0305819.s001.docx]

**Supplementary information for:** Relative contribution of biomedical, demographic, and socioeconomic factors to COVID-19 vaccine receipt in rural India

Bethany F. Ferris^1^, Suganthi Balasubramanian^2^, Anuradha Rajamanickam^2^, Saravanan Munisankar^2^, Bindu Dasan^2^, P’ng Loke^3^, Subash Babu^2^, Goylette F. Chami^1*^

**Table of Contents**

[**Supplementary Methods:** 2](#_Toc139633527)

[**S1 Table:** Age-specific blood pressure cut-off values used to define HTN in children 3](#_Toc139633528)

[**S2 Table:** List of medical conditions specified by the Indian government as enabling vaccine priority 4](#_Toc139633529)

[**S3 Table:** Self-reported medical conditions listed under seventeen anatomical sites 5](#_Toc139633530)

[**S4 Table:** Sex-specific neck circumference cut-off values used to categorise adults into BMI categories 7](#_Toc139633531)

[**S5 Table:** Determinants of vaccine receipt (univariable regression model) 8](#_Toc139633532)

[**S6 Table:** Determinants of vaccine receipt (multivariable regression model) 10](#_Toc139633533)

[**S7 Table:** Determinants of timing to vaccine receipt (univariable regression model) 11](#_Toc139633534)

[**S8 Table:** Determinants of timing to vaccine receipt (multivariable regression model) 13](#_Toc139633535)

[**S9 Table:** Participant characteristics split by vaccine eligibility. 14](#_Toc139633536)

[**S10 Table:** Determinants of vaccine receipt in all surveyed participants (univariable regression model) 16](#_Toc139633537)

[**S11 Table:** Determinants of vaccine receipt in all surveyed participants (multivariable regression model) 18](#_Toc139633538)

[**S12 Table:** Determinants of vaccine receipt using current medical condition (univariable regression model) 20](#_Toc139633539)

[**S13 Table:** Determinants of vaccine receipt using current medical condition (multivariable regression model) 22](#_Toc139633540)

## **Supplementary Methods:**

COVID-19 symptoms included: pneumonia, fever, chills, dyspnoea, dry persistent cough, diarrhoea, nausea or vomiting, abdominal pain, loss of appetite, headache, confusion, vertigo, new loss of taste, new loss of smell, sore throat, joint pain, new muscle or body aches, chest pain, fatigue, general weakness, rhinorrhoea, or depression.

COVID-19 related medications included: inotropes, anti-helminthics, corticosteroids, Janus kinase (JAK) inhibitors, remdesivir, tocilizumab, hydroxychloroquine, or azithromycin.

**Adults**

- Body mass index (BMI) categories included normal weight (BMI: 18.5-22.9), underweight (BMI≤18.5), overweight (BMI: 23-24.9), obese (BMI≥25). BMI categories were based upon South-Asian-specific BMI cut-offs [41].
- Raised HbA1c was defined as >6.5% [42].
- Hypertensive individuals were defined as systolic blood pressure ≥140mmHg or diastolic blood pressure ≥90mmHg [43].
- Abnormal HR was HR<60 or HR>100
- Abnormal RR was RR<12 or RR>20 [44].

**Children** (≤18 years)

- For BMI, age- and sex-specific cut-offs provided by the Indian Academy of Paediatrics were used to classify children, based on their BMI, into adult equivalent BMI categories [45]. These adult equivalent BMI categories were based on a BMI>25 as overweight and a BMI>27 as obese, thus were not the updated South-Asian-specific BMI cut-offs used for adults. There were 32 children classified using this method as normal weight (n=21), overweight (n=7) and obese (n=2) who may have been misclassified.
- Raised HbA1c was defined as >6.5% [42].
- Age-specific blood pressure cut-offs were used to classify children as hypertensive (File S1, S1 Table) [46].
- Abnormal HR was classified according to age: for children aged 12 years (HR<70 or HR>119) and children aged 13-17 years (HR<70 or HR>99) [47]
- Abnormal RR was classified as children aged 12 years (RR<20 or RR>29) and children aged 13-17 years (RR<15 or RR>25) [47]

## **S1 Table:** Age-specific blood pressure cut-off values used to define HTN in children

| Age | Systolic Blood Pressure (SBP) (mmHg) | Diastolic Blood Pressure (DBP) (mmHg) |
| --- | --- | --- |
| 12 | >112 | >74 |
| 13 | >119 | >79 |
| 14 | >119 | >79 |
| 15 | >119 | >79 |
| 16 | >119 | >79 |
| 17 | >119 | >79 |

Children were classified as hypertensive if the SBP or DBP were greater than the cut-off value.

## **S2 Table:** List of medical conditions specified by the Indian government as enabling vaccine priority


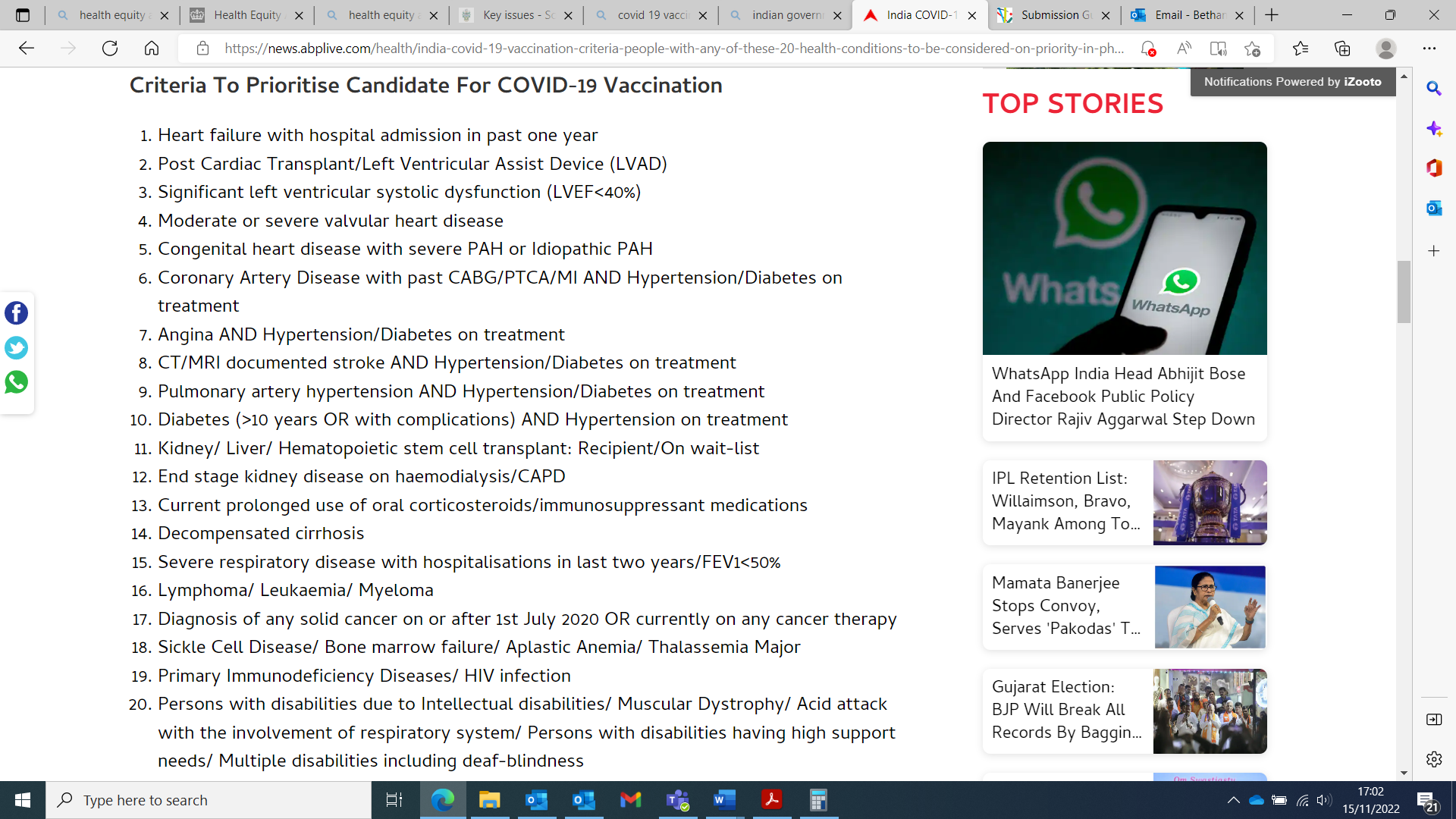


## **S3 Table:** Self-reported medical conditions listed under seventeen anatomical sites

| Anatomical Site | Overall number (%) | Number currently affected (% of all affected) | Specific conditions |
| --- | --- | --- | --- |
| Infectious | 9 | 1 | TB (n=8), HIV (n=1) |
| Cardiovascular | 74 | 38 | HTN (n=67), IHD (n=3), PIH (n=2), peripartum HF (n=1), VSD (n=1), CABG (n=1) |
| Dermatological | 25 | 14 | Tinea (n=12), scabies (n=1), vitiligo (n=2), psoriasis (n=1), SD (n=1), eczema (n=1), allergic dermatitis (n=3), chickenpox (n=1), herpes zoster (n=1), MM (n=1) |
| Digestive | 14 | 1 | PUD (n=10), oesophageal varices (n=1), appendicectomy (n=1), cholecystectomy (n=1), haemorrhoids + surgery (n=1) |
| Ear | 14 | 8 | Congenital hearing loss (n=2), loss of hearing post-trauma (n=1), hard of hearing (n=8), myringoplasty (n=1), allergic rhinitis (n=1), chronic OM (n=1) |
| Eye | 55 | 10 | Cataract (n=39), myopia/refractory error (n=11), proptosis (n=1), left superior oblique nerve palsy (n=1), injury and LOV (n=1), opted for dacrocystitis (n=1), RP (n=1) |
| Endocrine | 109 | 62 | DM (n=88), hypothyroidism (n=21), GDM (n=2) |
| Lymphatic | 1 | 0 | Unknown (n=1) |
| Respiratory | 15 | 6 | Asthma (n=10), allergic bronchitis (n=2), COPD (n=1), productive cough (n=1), bronchitis (n=1) |
| Genitourinary | 33 | 1 | Hysterectomy (n=23), fibroids (n=12), DUB (n=6), cervical cancer (n=1), ectopic (n=1), renal calculi (n=6), hydrocele (n=1), pyelonephritis post DJ stenting (n=1) |
| Immune | 2 | 1 | RA (n=2) |
| Nervous | 5 | 1 | Epilepsy (n=3), post-polio right leg (n=1), hemiplegia (n=1) |
| Skeletal | 23 | 4 | OA (n=6), degenerative spinal disease (n=4), RA (n=2), injury (n=5), non-specific pain (n=2), polio left limb (n=1), unclear (n=1), calcaneal spur (n=1) |
| Neoplasm | 1 | 0 | Uterine cancer |
| Psychological | 3 | 2 | Depression (n=1), mood disorder (n=1), tics (n=1) |
| Other | 1 | 1 | Unknown (n=1) |

CABG=coronary artery bypass graft, COPD=chronic obstructive pulmonary disease, DM=diabetes mellitus, DUB=dysfunctional uterine bleeding, GDM=gestational diabetes, HIV=human immunodeficiency virus, HTN=hypertension, IHD=ischaemic heart disease, MM=, OA=osteoarthritis, OM=otitis media, PIH=pregnancy induced hypertension, PUD=peptic ulcer disease, RA=rheumatoid arthritis, RP=retinitis pigmentosa, TB=tuberculosis, VSD=ventricular septal defect.

## **S4 Table:** Sex-specific neck circumference cut-off values used to categorise adults into BMI categories

| BMI Category | NC (cm) (male) | NC (cm) (female) | BMI (kg/m2) used to denote BMI category |
| --- | --- | --- | --- |
| Normal weight | - | - | - |
| Underweight | - | - | - |
| Overweight | >37.4 | >33.0 | >25 |
| Obese | >39.1 | >34.3 | >30 |

NC=neck circumference

## **S5 Table:** Determinants of vaccine receipt (univariable regression model)

| **Determinants** | OR | SE | 95% CI LL | 95% CI UL | LR chi^2^ p-value |
| --- | --- | --- | --- | --- | --- |
| **Demographics** |  |  |  |  |  |
| Age category (years) |  |  |  |  | 0.77 |
| <18 | 0.80 | 0.30 | 0.38 | 1.67 | 0.55 |
| 45-60 | 0.84 | 0.16 | 0.58 | 1.21 | 0.34 |
| >60 | 0.87 | 0.26 | 0.49 | 1.55 | 0.64 |
| Male | 1.15 | 0.19 | 0.83 | 1.60 | 0.41 |
| Majority tribe (Tamil) | 1.16 | 0.23 | 0.78 | 1.70 | 0.47 |
| Married* | 0.68 | 0.13 | 0.47 | 0.98 | 0.03 |
| Educational attainment* |  |  |  |  | 0.00 |
| Primary | 0.42 | 0.14 | 0.21 | 0.81 | 0.01 |
| High | 0.39 | 0.11 | 0.22 | 0.69 | 0.00 |
| Secondary | 0.92 | 0.28 | 0.51 | 1.68 | 0.79 |
| Senior Secondary | 1.09 | 0.40 | 0.54 | 2.23 | 0.81 |
| University | 1.27 | 0.40 | 0.69 | 2.35 | 0.45 |
| Post-graduate | 0.97 | 0.48 | 0.36 | 2.58 | 0.95 |
| Occupation group* |  |  |  |  | 0.00 |
| Retired or unemployed | 0.34 | 0.13 | 0.17 | 0.71 | 0.00 |
| Housewife | 0.21 | 0.06 | 0.12 | 0.37 | 0.00 |
| Student | 0.67 | 0.27 | 0.31 | 1.45 | 0.31 |
| Self-employed agricultural | 0.54 | 0.17 | 0.29 | 1.01 | 0.05 |
| Self-employed non-agricultural | 0.26 | 0.07 | 0.15 | 0.45 | 0.00 |
| Rural employment scheme | 1.65 | 0.95 | 0.53 | 5.09 | 0.38 |
| **SES** |  |  |  |  |  |
| Private healthcare use | 0.76 | 0.14 | 0.54 | 1.08 | 0.13 |
| Household land ownership* | 4.67 | 1.26 | 2.75 | 7.93 | 0.00 |
| Household PAN card ownership* | 1.78 | 0.31 | 1.26 | 2.51 | 0.00 |
| Household ration card ownership* | 3.32 | 1.23 | 1.61 | 6.86 | 0.00 |
| **WASH** |  |  |  |  |  |
| Improved Sanitation* | 0.38 | 0.10 | 0.23 | 0.63 | 0.00 |
| Improved Hygiene | 1.74 | 0.69 | 0.80 | 3.79 | 0.17 |
| **Biomedical** |  |  |  |  |  |
| Priority medical condition* | 0.34 | 0.15 | 0.14 | 0.82 | 0.02 |
| BMI category* |  |  |  |  | 0.03 |
| Underweight | 0.91 | 0.30 | 0.47 | 1.76 | 0.78 |
| Overweight | 1.78 | 0.49 | 1.04 | 3.06 | 0.04 |
| Obese | 0.88 | 0.18 | 0.59 | 1.30 | 0.52 |
| Raised HbA1c* | 0.70 | 0.14 | 0.47 | 1.04 | 0.08 |
| Hypertension | 0.83 | 0.16 | 0.57 | 1.22 | 0.35 |
| Abnormal heart rate* | 0.62 | 0.17 | 0.36 | 1.08 | 0.09 |
| Extensive travel* | 2.61 | 0.46 | 1.84 | 3.69 | 0.00 |
| COVID-19 contact* | 0.35 | 0.15 | 0.16 | 0.79 | 0.01 |
| COVID-19 test taken* | 1.89 | 0.42 | 1.22 | 2.93 | 0.00 |
| Positive COVID-19 PCR result* | 0.33 | 0.19 | 0.10 | 1.05 | 0.06 |
| COVID-19 symptoms | 0.62 | 0.20 | 0.34 | 1.15 | 0.14 |
| COVID-19 medications taken* | 0.32 | 0.16 | 0.12 | 0.85 | 0.02 |
| Day of data collection* | 1.01 | 0.00 | 1.00 | 1.01 | 0.00 |

BMI=body mass index; CI=confidence interval; HbA1c=glycated haemoglobin; LL=lower limit; LR=likelihood ratio; OR=odds ratio; PAN=permanent account number; PCR=polymerase chain reaction; SES=socioeconomic status; SE=standard error; UL=upper limit; WASH=water, sanitation, and hygiene.

*LR chi^2^ p-value<0.10

## **S6 Table:** Determinants of vaccine receipt (multivariable regression model)

| **Determinants** | AOR | Robust SE | 95% CI LL | 95% CI UL | p-value |
| --- | --- | --- | --- | --- | --- |
| **Demographic** |  |  |  |  |  |
| Married | 1.02 | 0.26 | 0.62 | 1.67 | 0.94 |
| Educational attainment |  |  |  |  | 0.15 |
| Primary | 0.58 | 0.23 | 0.27 | 1.25 | 0.16 |
| High | 0.51 | 0.18 | 0.25 | 1.04 | 0.06 |
| Secondary | 0.78 | 0.28 | 0.38 | 1.58 | 0.49 |
| Senior Secondary | 1.10 | 0.43 | 0.52 | 2.36 | 0.80 |
| University | 1.06 | 0.38 | 0.53 | 2.13 | 0.87 |
| Post-graduate | 1.51 | 0.97 | 0.43 | 5.33 | 0.52 |
| Occupation group |  |  |  |  | 0.03 |
| Retired or unemployed | 0.54 | 0.25 | 0.22 | 1.34 | 0.18 |
| Housewife | 0.35 | 0.12 | 0.19 | 0.67 | <0.01 |
| Student | 0.57 | 0.30 | 0.20 | 1.60 | 0.29 |
| Self-employed agricultural | 0.69 | 0.28 | 0.31 | 1.52 | 0.36 |
| Self-employed non-agricultural | 0.41 | 0.13 | 0.22 | 0.76 | <0.01 |
| Rural employment scheme | 0.73 | 0.48 | 0.20 | 2.62 | 0.63 |
| **SES** |  |  |  |  |  |
| Household land ownership | 1.70 | 0.65 | 0.80 | 3.61 | 0.17 |
| Household PAN card ownership | 2.15 | 0.54 | 1.32 | 3.52 | <0.01 |
| Household ration card ownership | 3.02 | 0.86 | 1.72 | 5.29 | <0.01 |
| Improved Sanitation | 1.16 | 0.43 | 0.56 | 2.39 | 0.69 |
| **Biomedical** |  |  |  |  |  |
| Priority medical condition | 0.44 | 0.21 | 0.17 | 1.10 | 0.08 |
| BMI category |  |  |  |  | 0.10 |
| Underweight | 1.18 | 0.45 | 0.56 | 2.48 | 0.66 |
| Overweight* | 2.12 | 0.67 | 1.14 | 3.94 | 0.02 |
| Obese | 1.06 | 0.26 | 0.66 | 1.71 | 0.80 |
| Raised HbA1c | 0.94 | 0.25 | 0.56 | 1.60 | 0.83 |
| Abnormal heart rate | 0.63 | 0.19 | 0.36 | 1.13 | 0.12 |
| Extensive travel | 1.17 | 0.31 | 0.70 | 1.96 | 0.56 |
| COVID-19 contact | 0.28 | 0.22 | 0.06 | 1.30 | 0.11 |
| COVID-19 test taken | 1.69 | 0.50 | 0.94 | 3.02 | 0.08 |
| Positive COVID-19 PCR result | 0.62 | 0.63 | 0.09 | 4.48 | 0.64 |
| COVID-19 medications taken | 0.52 | 0.37 | 0.13 | 2.09 | 0.36 |
| Day of data collection | 1.00 | 0.00 | 1.00 | 1.01 | <0.01 |
| Constant | 0.00 | 0.00 | 0.00 | 0.00 | 0.00 |

N= 650 participants. 5-fold cross-validated mean area under the receiver operating curve=0.737 (SD 0.030). Robust standard errors are clustered at the household level. There are 262 clusters.

AOR=adjusted odds ratio; BMI=body mass index, CI=confidence interval; HbA1c=glycated haemoglobin; LL=lower limit; PAN=permanent account number; PCR=polymerase chain reaction; SE=standard error; UL=upper limit.

## **S7 Table:** Determinants of timing to vaccine receipt (univariable regression model)

| Determinants | OR | SE | 95% CI LL | 95% CI UL | LR chi^2^ p-value |
| --- | --- | --- | --- | --- | --- |
| **Demographics** |  |  |  |  |  |
| Age category (years)* |  |  |  |  | 0.00 |
| <18 | 24.36 | 10.32 | 10.61 | 55.90 | 0.00 |
| 45-60 | 3.79 | 1.14 | 2.10 | 6.85 | 0.00 |
| >60 | 0.96 | 0.61 | 0.28 | 3.36 | 0.95 |
| Male | 1.32 | 0.33 | 0.82 | 2.15 | 0.25 |
| Majority tribe (Tamil) | 1.19 | 0.36 | 0.65 | 2.16 | 0.57 |
| Married* | 0.51 | 0.13 | 0.31 | 0.83 | 0.01 |
| Educational attainment |  |  |  |  | 0.17 |
| Primary | 1.17 | 0.62 | 0.41 | 3.33 | 0.76 |
| High | 0.77 | 0.37 | 0.30 | 1.98 | 0.59 |
| Secondary | 1.35 | 0.60 | 0.57 | 3.21 | 0.49 |
| Senior Secondary | 1.88 | 0.90 | 0.73 | 4.82 | 0.19 |
| University | 1.23 | 0.55 | 0.51 | 2.93 | 0.65 |
| Post-graduate | 3.52 | 1.99 | 1.17 | 10.64 | 0.03 |
| Occupation group* |  |  |  |  | 0.00 |
| Retired or unemployed | 0.61 | 0.32 | 0.22 | 1.70 | 0.34 |
| Housewife | 0.38 | 0.16 | 0.17 | 0.87 | 0.02 |
| Student | 3.00 | 1.10 | 1.46 | 6.17 | 0.00 |
| Self-employed agricultural | 0.52 | 0.22 | 0.23 | 1.17 | 0.11 |
| Self-employed non-agricultural | 0.37 | 0.15 | 0.16 | 0.83 | 0.02 |
| Rural employment scheme | 0.14 | 0.15 | 0.02 | 1.11 | 0.06 |
| **SES** |  |  |  |  |  |
| Private healthcare use | 1.36 | 0.35 | 0.83 | 2.25 | 0.23 |
| Household land ownership* | 2.24 | 1.19 | 0.79 | 6.33 | 0.09 |
| Household PAN card ownership* | 2.18 | 0.68 | 1.19 | 4.01 | 0.01 |
| Household ration card ownership | 2.02 | 1.50 | 0.47 | 8.63 | 0.30 |
| **WASH** |  |  |  |  |  |
| Improved Sanitation | 0.77 | 0.23 | 0.43 | 1.37 | 0.38 |
| Improved Hygiene | 3.52 | 3.62 | 0.47 | 26.35 | 0.14 |
| **Biomedical** |  |  |  |  |  |
| Priority medical condition | 0.80 | 0.60 | 0.18 | 3.49 | 0.76 |
| BMI category* |  |  |  |  | 0.03 |
| Underweight | 0.69 | 0.36 | 0.25 | 1.90 | 0.47 |
| Overweight | 1.40 | 0.46 | 0.74 | 2.65 | 0.30 |
| Obese | 0.56 | 0.17 | 0.31 | 1.02 | 0.06 |
| Raised HbA1c | 0.94 | 0.29 | 0.52 | 1.72 | 0.84 |
| Hypertension | 1.41 | 0.38 | 0.83 | 2.41 | 0.21 |
| Abnormal heart rate | 1.27 | 0.51 | 0.58 | 2.81 | 0.56 |
| Extensive travel | 1.34 | 0.37 | 0.78 | 2.29 | 0.29 |
| COVID-19 contact | 1.97 | 1.02 | 0.72 | 5.42 | 0.22 |
| COVID-19 test taken | 0.97 | 0.29 | 0.54 | 1.74 | 0.92 |
| Positive COVID-19 PCR result | 0.69 | 0.72 | 0.09 | 5.42 | 0.71 |
| COVID-19 symptoms | 1.45 | 0.62 | 0.62 | 3.37 | 0.41 |
| COVID-19 medications taken | 1.02 | 0.78 | 0.23 | 4.54 | 0.98 |
| Day of data collection** | 1.00 | 0.00 | 1.00 | 1.00 | 0.87 |

BMI=body mass index; CI=confidence interval; HbA1c=glycated haemoglobin; LL=lower limit; LR=likelihood ratio; OR=odds ratio; PAN=permanent account number; PCR=polymerase chain reaction; SES=socioeconomic status; SE=standard error; UL=upper limit; WASH=water, sanitation, and hygiene.

*LR chi^2^ p-value<0.10

**LR chi^2^ p-value>0.10 but included in fully adjusted model as a confounding factor

## **S8 Table:** Determinants of timing to vaccine receipt (multivariable regression model)

| **Determinants** | AOR | Robust  SE | 95% CI LL | 95% CI  UL | p-value |
| --- | --- | --- | --- | --- | --- |
| **Demographic** |  |  |  |  |  |
| Age category (years) |  |  |  |  | <0.01 |
| ≤18 | 17.74 | 11.33 | 5.07 | 62.03 | <0.01 |
| 45-60 | 5.51 | 1.97 | 2.74 | 11.10 | <0.01 |
| >60 | 1.04 | 0.70 | 0.27 | 3.93 | 0.96 |
| Married | 0.74 | 0.27 | 0.36 | 1.53 | 0.42 |
| Occupation group |  |  |  |  | 0.02 |
| Retired or unemployed | 0.56 | 0.36 | 0.16 | 2.00 | 0.37 |
| Housewife | 0.35 | 0.16 | 0.14 | 0.86 | 0.02 |
| Student | 0.94 | 0.51 | 0.32 | 2.74 | 0.91 |
| Self-employed agricultural | 0.32 | 0.15 | 0.13 | 0.79 | <0.01 |
| Self-employed non-agricultural | 0.31 | 0.13 | 0.14 | 0.71 | <0.01 |
| Rural employment scheme | 0.14 | 0.15 | 0.02 | 1.05 | 0.06 |
| **SES** |  |  |  |  |  |
| Household land ownership | 1.67 | 1.02 | 0.50 | 5.54 | 0.40 |
| Household PAN card ownership | 1.87 | 0.60 | 1.00 | 3.52 | 0.05 |
| **Biomedical** |  |  |  |  |  |
| BMI category |  |  |  |  | 0.05 |
| Underweight | 1.41 | 0.83 | 0.44 | 4.48 | 0.56 |
| Overweight | 2.20 | 0.84 | 1.04 | 4.66 | 0.04 |
| Obese | 0.94 | 0.35 | 0.45 | 1.94 | 0.86 |
| Day of data collection | 1.00 | 0.00 | 0.99 | 1.00 | 0.09 |
| Constant | 3.37E+21 | 1.06E+23 | 4.87E-06 | 2.33E+48 | 0.12 |

N= 647 participants. 5-fold cross-validated mean area under the receiver operating curve=0.663 (SD:0.082). Robust standard errors are clustered at the household level. There are 262 clusters.

AOR=adjusted odds ratio; BMI=body mass index, CI=confidence interval; LL=lower limit; PAN=permanent account number; SE=standard error; UL=upper limit.

## **S9 Table:** Participant characteristics split by vaccine eligibility.

|  | Total (n=743) | Ineligible (n=93) | Eligible (n=650) | Chi^2^ p-value |
| --- | --- | --- | --- | --- |
| **Demographic** |  |  |  |  |
| Age category (years)* |  |  |  | <0.01 |
| <18 | 127 (17.1) | 93 (100.0) | 34 (5.2) |  |
| 19-44 | 360 (48.5) | 0 (0.0) | 360 (55.4) |  |
| 45-60 | 196 (26.4) | 0 (0.0) | 196 (30.2) |  |
| >60 | 60 (8.1) | 0 (0.0) | 60 (9.2) |  |
| Male* | 360 (48.5) | 54 (58.1) | 306 (47.1) | <0.05 |
| Majority tribe (Tamil) | 575 (77.4) | 73 (78.5) | 502 (77.2) | 0.79 |
| Married* | 445 (59.9) | 0 (0.0) | 445 (68.5) | <0.01 |
| Educational attainment* |  |  |  | <0.01 |
| No formal schooling | 95 (12.8) | 0 (0.0) | 95 (14.6) |  |
| Primary | 85 (11.4) | 20 (21.5) | 65 (10.0) |  |
| High | 166 (22.3) | 32 (34.4) | 134 (20.6) |  |
| Secondary | 147 (19.8) | 18 (19.4) | 129 (19.8) |  |
| Senior Secondary | 84 (11.3) | 15 (16.1) | 69 (10.6) |  |
| University | 140 (18.8) | 8 (8.6) | 132 (20.3) |  |
| Post-graduate | 26 (3.5) | 0 (0.0) | 26 (4.0) |  |
| Occupation group* |  |  |  | <0.01 |
| Salaried | 144 (19.4) | 0 (0.0) | 144 (22.2) |  |
| Retired or unemployed | 49 (6.6) | 0 (0.0) | 49 (7.5) |  |
| Housewife | 132 (17.8) | 0 (0.0) | 132 (20.3) |  |
| Student | 145 (19.5) | 93 (100.0) | 52 (8.0) |  |
| Self-employed agricultural | 100 (13.5) | 0 (0.0) | 100 (15.4) |  |
| Self-employed non-agricultural | 136 (18.3) | 0 (0.0) | 136 (20.9) |  |
| Rural Employment Scheme | 37 (5.0) | 0 (0.0) | 37 (5.7) |  |
| **SES** |  |  |  |  |
| Private healthcare use | 233 (31.4) | 31 (33.3) | 202 (31.1) | 0.66 |
| Household land ownership* | 652 (87.8) | 70 (75.3) | 582 (89.5) | <0.01 |
| Household electricity supply | 741 (99.7) | 93 (100) | 648 (99.7) |  |
| Household PAN card ownership | 502 (67.6) | 58 (62.4) | 444 (68.3) | 0.25 |
| Household ration card ownership* | 710 (95.6) | 92 (98.9) | 618 (95.1) | 0.09 |
| **WASH** |  |  |  |  |
| Improved sanitation | 602 (81.0) | 74 (79.6) | 528 (81.2) | 0.70 |
| Protected water source | 740 (99.6) | 93 (100) | 647 (99.5) | 0.51 |
| Improved hygiene | 709 (95.4) | 86 (92.5) | 623 (95.8) | 0.15 |
| **Biomedical** |  |  |  |  |
| Recent hospital admission | 8 (1.1) | 0 (0) | 8 (1.2) | 0.28 |
| Priority medical condition* | 21 (2.8) | 0 (0.0) | 21 (3.2) | 0.08 |
| BMI category* |  |  |  | <0.01 |
| Normal weight | 224 (30.1) | 55 (59.1) | 169 (26.0) |  |
| Underweight | 61 (8.2) | 9 (9.7) | 52 (8.0) |  |
| Overweight | 142 (19.1) | 20 (21.5) | 122 (18.8) |  |
| Obese | 316 (42.5) | 9 (9.7) | 307 (47.2) |  |
| Raised HbA1c* | 139 (18.7) | 3 (3.2) | 136 (20.9) | <0.01 |
| Hypertension | 170 (22.9) | 16 (17.2) | 154 (23.7) | 0.16 |
| Abnormal heart rate | 68 (9.2) | 11 (11.8) | 57 (8.8) | 0.34 |
| Abnormal respiratory rate* | 39 (5.2) | 24 (25.8) | 15 (2.3) | <0.01 |
| Travel history* | 475 (63.9) | 33 (35.5) | 442 (68.0) | <0.01 |
| COVID-19 contact* | 32 (4.3) | 7 (7.5) | 25 (3.8) | 0.10 |
| Household in a containment zone* | 8 (1.1) | 3 (3.2) | 5 (0.8) | 0.03 |
| COVID-19 test taken* | 145 (19.5) | 4 (4.3) | 141 (21.7) | <0.01 |
| Positive COVID-19 PCR result | 12 (1.6) | 0 (0.0) | 12 (1.8) | 0.19 |
| COVID-19 symptoms | 53 (7.1) | 8 (8.6) | 45 (6.9) | 0.56 |
| COVID-19 medications taken | 19 (2.6) | 2 (2.2) | 17 (2.6) | 0.79 |

BMI=body mass index; HbA1c=glycated haemoglobin; PAN=permanent account number; PCR=polymerase chain reaction; SES=socioeconomic status; WASH=water, sanitation, and hygiene.

*LR chi^2^ p-value<0.10

## **S10 Table:** Determinants of vaccine receipt in all surveyed participants (univariable regression model)

| **Determinants** | OR | SE | | 95% CI LL | 95% CI UL | LR chi^2^ p-value |
| --- | --- | --- | --- | --- | --- | --- |
| **Demographic** |  |  |  | |  |  |
| Age category (years)* |  |  |  | |  | <0.01 |
| <18 | 0.10 | 0.03 | 0.06 | | 0.17 | <0.01 |
| 45-60 | 0.84 | 0.16 | 0.58 | | 1.21 | 0.34 |
| >60 | 0.87 | 0.26 | 0.49 | | 1.55 | 0.64 |
| Male | 0.95 | 0.14 | 0.71 | | 1.28 | 0.75 |
| Majority tribe (Tamil) | 1.11 | 0.20 | 0.79 | | 1.58 | 0.54 |
| Married* | 1.79 | 0.27 | 1.33 | | 2.42 | <0.01 |
| Educational attainment* |  |  |  | |  | <0.01 |
| Primary | 0.25 | 0.08 | 0.13 | | 0.47 | <0.01 |
| High | 0.26 | 0.07 | 0.15 | | 0.45 | <0.01 |
| Secondary | 0.65 | 0.19 | 0.37 | | 1.15 | 0.14 |
| Senior Secondary | 0.58 | 0.19 | 0.31 | | 1.09 | 0.09 |
| University | 0.99 | 0.30 | 0.55 | | 1.80 | 0.99 |
| Post-graduate | 0.97 | 0.48 | 0.36 | | 2.58 | 0.95 |
| Occupation group* |  |  |  | |  | <0.01 |
| Retired or unemployed | 0.34 | 0.13 | 0.17 | | 0.71 | <0.01 |
| Housewife | 0.21 | 0.06 | 0.12 | | 0.37 | <0.01 |
| Student | 0.08 | 0.02 | 0.05 | | 0.14 | <0.01 |
| Self-employed agricultural | 0.54 | 0.17 | 0.29 | | 1.01 | 0.05 |
| Self-employed non-agricultural | 0.26 | 0.07 | 0.15 | | 0.45 | <0.01 |
| Rural employment scheme | 1.65 | 0.95 | 0.53 | | 5.09 | 0.38 |
| **SES** |  |  |  | |  |  |
| Private healthcare use* | 0.77 | 0.12 | 0.56 | | 1.05 | 0.10 |
| Household land ownership* | 5.05 | 1.27 | 3.09 | | 8.27 | <0.01 |
| Household PAN card ownership* | 1.74 | 0.28 | 1.28 | | 2.38 | <0.01 |
| Household ration card ownership* | 2.38 | 0.87 | 1.16 | | 4.85 | 0.02 |
| **WASH** |  |  |  | |  |  |
| Improved Sanitation* | 0.48 | 0.10 | 0.32 | | 0.72 | <0.01 |
| Improved Hygiene* | 1.94 | 0.69 | 0.97 | | 3.88 | 0.06 |
| **Biomedical** |  |  |  | |  |  |
| Priority medical condition | 0.49 | 0.22 | 0.21 | | 1.19 | 0.11 |
| BMI category* |  |  |  | |  | <0.01 |
| Underweight | 1.19 | 0.35 | 0.68 | | 2.11 | 0.54 |
| Overweight | 2.04 | 0.46 | 1.32 | | 3.17 | <0.01 |
| Obese | 1.59 | 0.28 | 1.12 | | 2.25 | <0.01 |
| Raised HbA1c | 1.04 | 0.20 | 0.71 | | 1.51 | 0.86 |
| Hypertension | 0.95 | 0.17 | 0.67 | | 1.35 | 0.78 |
| Abnormal heart rate* | 0.59 | 0.15 | 0.36 | | 0.96 | <0.01 |
| Abnormal respiratory rate* | 0.14 | 0.08 | 0.05 | | 0.42 | <0.01 |
| Extensive travel* | 3.23 | 0.51 | 2.37 | | 4.42 | <0.01 |
| COVID-19 contact* | 0.34 | 0.13 | 0.16 | | 0.71 | <0.01 |
| COVID-19 test taken* | 2.48 | 0.52 | 1.64 | | 3.76 | <0.01 |
| Positive COVID-19 PCR result | 0.48 | 0.28 | 0.15 | | 1.51 | 0.20 |
| COVID-19 symptoms | 0.63 | 0.18 | 0.36 | | 1.10 | 0.10 |
| COVID-19 medications taken* | 0.38 | 0.18 | 0.15 | | 0.98 | 0.04 |
| Day of data collection* | 1.01 | 0.00 | 1.00 | | 1.01 | <0.01 |
| Household in containment zone | 0.67 | 0.48 | 0.17 | | 2.70 | 0.58 |

BMI=body mass index; CI=confidence interval; HbA1c=glycated haemoglobin; LL=lower limit; LR=likelihood ratio; OR=odds ratio; PAN=permanent account number; PCR=polymerase chain reaction; SES=socioeconomic status; SE=standard error; UL=upper limit; WASH=water, sanitation, and hygiene.

*LR chi^2^ p-value<0.10

## **S11 Table:** Determinants of vaccine receipt in all surveyed participants (multivariable regression model)

| **Determinants** | AOR | Robust  SE | 95% CI  LL | 95% CI  UL | p-value |
| --- | --- | --- | --- | --- | --- |
| **Demographic** |  |  |  |  |  |
| Age category (years) |  |  |  |  | <0.01 |
| ≤18 | 0.03 | 0.02 | 0.01 | 0.11 | <0.01 |
| 45-60 | 1.79 | 0.47 | 1.08 | 2.99 | 0.03 |
| >60 | 2.40 | 1.02 | 1.04 | 5.52 | 0.04 |
| Married | 0.78 | 0.21 | 0.46 | 1.34 | 0.37 |
| Educational attainment |  |  |  |  | <0.01 |
| Primary | 0.55 | 0.21 | 0.26 | 1.18 | 0.13 |
| High | 0.75 | 0.29 | 0.35 | 1.59 | 0.45 |
| Secondary | 1.54 | 0.62 | 0.70 | 3.39 | 0.28 |
| Senior Secondary | 2.20 | 0.94 | 0.95 | 5.10 | 0.06 |
| University | 1.97 | 0.79 | 0.90 | 4.31 | 0.09 |
| Post-graduate | 2.67 | 1.75 | 0.74 | 9.66 | 0.13 |
| Occupation group |  |  |  |  | 0.10 |
| Retired or unemployed | 0.51 | 0.23 | 0.21 | 1.26 | 0.14 |
| Housewife | 0.40 | 0.14 | 0.20 | 0.78 | <0.01 |
| Student | 1.21 | 0.66 | 0.41 | 3.55 | 0.73 |
| Self-employed agricultural | 0.62 | 0.27 | 0.27 | 1.45 | 0.27 |
| Self-employed non-agricultural | 0.46 | 0.16 | 0.23 | 0.92 | 0.03 |
| Rural employment scheme | 0.49 | 0.34 | 0.13 | 1.89 | 0.30 |
| **SES** |  |  |  |  |  |
| Private healthcare use | 0.94 | 0.25 | 0.57 | 1.57 | 0.83 |
| Household land ownership | 1.59 | 0.55 | 0.80 | 3.14 | 0.19 |
| Household PAN card ownership | 2.16 | 0.53 | 1.33 | 3.49 | <0.01 |
| Household ration card ownership | 3.00 | 0.82 | 1.75 | 5.11 | <0.01 |
| **WASH** |  |  |  |  |  |
| Improved sanitation | 1.03 | 0.36 | 0.52 | 2.05 | 0.94 |
| Improved hygiene | 1.17 | 0.62 | 0.41 | 3.31 | 0.77 |
| **Biomedical** |  |  |  |  |  |
| BMI category |  |  |  |  | 0.05 |
| Underweight | 0.83 | 0.29 | 0.42 | 1.63 | 0.58 |
| Overweight | 1.97 | 0.59 | 1.10 | 3.53 | 0.02 |
| Obese | 0.95 | 0.22 | 0.60 | 1.51 | 0.84 |
| Abnormal heart rate | 0.58 | 0.17 | 0.32 | 1.04 | 0.07 |
| Abnormal respiratory rate | 0.60 | 0.56 | 0.10 | 3.75 | 0.59 |
| Extensive travel | 1.24 | 0.29 | 0.78 | 1.96 | 0.36 |
| COVID-19 contact | 0.27 | 0.18 | 0.07 | 0.98 | 0.05 |
| COVID-19 test taken | 1.44 | 0.42 | 0.81 | 2.54 | 0.21 |
| COVID-19 medications taken | 0.29 | 0.19 | 0.08 | 1.08 | 0.06 |
| Day of data collection | 1.01 | 0.00 | 1.00 | 1.01 | <0.01 |
| Constant | 0.00 | 0.00 | 0.00 | 0.00 | 0.00 |

N= 743 participants. 5-fold cross-validated mean area under the receiver operating curve=0.802 (SD: 0.046). Robust standard errors are clustered at the household level. There are 262 clusters.

AOR=adjusted odds ratio; BMI=body mass index, CI=confidence interval; LL=lower limit; PAN=permanent account number; SES=socioeconomic status; SE=standard error; UL=upper limit; WASH=water, sanitation, and hygiene.

## **S12 Table:** Determinants of vaccine receipt using current medical condition (univariable regression model)

| **Determinants** | OR | SE | 95% CI LL | 95% CI UL | LR chi^2^ p-value |
| --- | --- | --- | --- | --- | --- |
| **Demographic** |  |  |  |  |  |
| Age category (years) |  |  |  |  | 0.77 |
| <18 | 0.80 | 0.30 | 0.38 | 1.67 | 0.55 |
| 45-60 | 0.84 | 0.16 | 0.58 | 1.21 | 0.34 |
| >60 | 0.87 | 0.26 | 0.49 | 1.55 | 0.64 |
| Male | 1.15 | 0.19 | 0.83 | 1.60 | 0.41 |
| Majority tribe (Tamil) | 1.16 | 0.23 | 0.78 | 1.70 | 0.47 |
| Married* | 0.68 | 0.13 | 0.47 | 0.98 | 0.03 |
| Educational attainment* |  |  |  |  | <0.01 |
| Primary | 0.42 | 0.14 | 0.21 | 0.81 | <0.01 |
| High | 0.39 | 0.11 | 0.22 | 0.69 | <0.01 |
| Secondary | 0.92 | 0.28 | 0.51 | 1.68 | 0.79 |
| Senior Secondary | 1.09 | 0.40 | 0.54 | 2.23 | 0.81 |
| University | 1.27 | 0.40 | 0.69 | 2.35 | 0.45 |
| Post-graduate | 0.97 | 0.48 | 0.36 | 2.58 | 0.95 |
| Occupation group* |  |  |  |  | <0.01 |
| Retired or unemployed | 0.34 | 0.13 | 0.17 | 0.71 | <0.01 |
| Housewife | 0.21 | 0.06 | 0.12 | 0.37 | <0.01 |
| Student | 0.67 | 0.27 | 0.31 | 1.45 | 0.31 |
| Self-employed agricultural | 0.54 | 0.17 | 0.29 | 1.01 | 0.05 |
| Self-employed non-agricultural | 0.26 | 0.07 | 0.15 | 0.45 | <0.01 |
| Rural employment scheme | 1.65 | 0.95 | 0.53 | 5.09 | 0.38 |
| **SES** |  |  |  |  |  |
| Private healthcare use | 0.76 | 0.14 | 0.54 | 1.08 | 0.13 |
| Household land ownership* | 4.67 | 1.26 | 2.75 | 7.93 | <0.01 |
| Household PAN card ownership* | 1.78 | 0.31 | 1.26 | 2.51 | <0.01 |
| Household ration card ownership* | 3.32 | 1.23 | 1.61 | 6.86 | <0.01 |
| **WASH** |  |  |  |  |  |
| Improved sanitation* | 0.38 | 0.10 | 0.23 | 0.63 | <0.01 |
| Improved hygiene | 1.74 | 0.69 | 0.80 | 3.79 | 0.17 |
| **Biomedical** |  |  |  |  |  |
| Current medical condition | 0.95 | 0.21 | 0.61 | 1.47 | 0.80 |
| BMI category* |  |  |  |  | 0.03 |
| Underweight | 0.91 | 0.30 | 0.47 | 1.76 | 0.78 |
| Overweight | 1.78 | 0.49 | 1.04 | 3.06 | 0.04 |
| Obese | 0.88 | 0.18 | 0.59 | 1.30 | 0.52 |
| Raised HbA1c* | 0.70 | 0.14 | 0.47 | 1.04 | 0.08 |
| Hypertension | 0.83 | 0.16 | 0.57 | 1.22 | 0.35 |
| Abnormal heart rate | 0.62 | 0.17 | 0.36 | 1.08 | 0.09 |
| Extensive travel* | 2.61 | 0.46 | 1.84 | 3.69 | <0.01 |
| COVID-19 contact* | 0.35 | 0.15 | 0.16 | 0.79 | <0.01 |
| COVID-19 test taken* | 1.89 | 0.42 | 1.22 | 2.93 | <0.01 |
| Positive COVID-19 PCR result* | 0.33 | 0.19 | 0.10 | 1.05 | 0.06 |
| COVID-19 symptoms | 0.62 | 0.20 | 0.34 | 1.15 | 0.14 |
| COVID-19 medications taken* | 0.32 | 0.16 | 0.12 | 0.85 | 0.02 |
| Day of data collection* | 1.01 | 0.00 | 1.00 | 1.01 | 0.00 |

BMI=body mass index; CI=confidence interval; HbA1c=glycated haemoglobin; LL=lower limit; LR=likelihood ratio; OR=odds ratio; PAN=permanent account number; PCR=polymerase chain reaction; SES=socioeconomic status; SE=standard error; UL=upper limit; WASH=water, sanitation, and hygiene.

*LR chi^2^ p-value<0.10

## **S13 Table:** Determinants of vaccine receipt using current medical condition (multivariable regression model)

| **Determinants** | AOR | Robust  SE | 95% CI LL | 95% CI  UL | p-value |
| --- | --- | --- | --- | --- | --- |
| **Demographic** |  |  |  |  |  |
| Married | 1.02 | 0.25 | 0.62 | 1.66 | 0.94 |
| Educational attainment |  |  |  |  | 0.16 |
| Primary | 0.60 | 0.24 | 0.28 | 1.30 | 0.19 |
| High | 0.53 | 0.19 | 0.26 | 1.07 | 0.08 |
| Secondary | 0.82 | 0.30 | 0.40 | 1.66 | 0.58 |
| Senior Secondary | 1.11 | 0.43 | 0.52 | 2.36 | 0.79 |
| University | 1.10 | 0.39 | 0.54 | 2.22 | 0.79 |
| Post-graduate | 1.57 | 1.01 | 0.45 | 5.52 | 0.48 |
| Occupation group |  |  |  |  | 0.03 |
| Retired or unemployed | 0.53 | 0.24 | 0.21 | 1.31 | 0.17 |
| Housewife | 0.35 | 0.11 | 0.19 | 0.66 | <0.01 |
| Student | 0.58 | 0.30 | 0.20 | 1.62 | 0.30 |
| Self-employed agricultural | 0.68 | 0.27 | 0.31 | 1.49 | 0.33 |
| Self-employed non-agricultural | 0.41 | 0.13 | 0.22 | 0.77 | <0.01 |
| Rural employment scheme | 0.72 | 0.47 | 0.20 | 2.61 | 0.62 |
| **SES** |  |  |  |  |  |
| Household land ownership | 1.68 | 0.64 | 0.79 | 3.53 | 0.18 |
| Household PAN card ownership | 2.10 | 0.52 | 1.29 | 3.43 | <0.01 |
| Household ration card ownership | 2.98 | 0.86 | 1.70 | 5.25 | <0.01 |
| **WASH** |  |  |  |  |  |
| Improved Sanitation | 1.15 | 0.42 | 0.56 | 2.36 | 0.70 |
| **Biomedical** |  |  |  |  |  |
| BMI category |  |  |  |  | 0.10 |
| Underweight | 1.16 | 0.44 | 0.55 | 2.42 | 0.70 |
| Overweight | 2.08 | 0.65 | 1.12 | 3.84 | 0.02 |
| Obese | 1.06 | 0.26 | 0.65 | 1.71 | 0.82 |
| Raised HbA1c | 0.85 | 0.22 | 0.52 | 1.40 | 0.52 |
| Abnormal heart rate | 0.62 | 0.18 | 0.35 | 1.09 | 0.10 |
| Extensive travel | 1.14 | 0.30 | 0.68 | 1.90 | 0.62 |
| COVID-19 contact | 0.28 | 0.22 | 0.06 | 1.31 | 0.11 |
| COVID-19 test taken | 1.71 | 0.51 | 0.96 | 3.05 | 0.07 |
| Positive COVID-19 PCR result | 0.48 | 0.44 | 0.08 | 2.85 | 0.42 |
| COVID-19 medications taken | 0.49 | 0.32 | 0.13 | 1.80 | 0.28 |
| Day of data collection | 1.00 | 0.00 | 1.00 | 1.01 | <0.01 |
| Constant | 0.00 | 0.00 | 0.00 | 0.00 | 0.00 |

N= 650 participants. 5-fold cross-validated mean area under the receiver operating curve=0.738 (SD:0.032). Robust standard errors are clustered at the household level. There are 262 clusters.

BMI=body mass index, HbA1c=glycated haemoglobin, PAN=permanent account number; PCR=polymerase chain reaction; SES=socioeconomic status; WASH=water, sanitation, and hygiene.
